# Pitfalls of bacterial pan-genome analysis approaches: a case study of *Mycobacterium tuberculosis* and two less clonal bacterial species

**DOI:** 10.1093/bioinformatics/btaf219

**Published:** 2025-05-08

**Authors:** Maximillian G Marin, Natalia Quinones-Olvera, Christoph Wippel, Mahboobeh Behruznia, Brendan M Jeffrey, Michael Harris, Brendon C Mann, Alex Rosenthal, Karen R Jacobson, Robin M Warren, Heng Li, Conor J Meehan, Maha R Farhat

**Affiliations:** Department of Biomedical Informatics, Harvard Medical School, Boston, MA 02115, United States; Department of Biomedical Informatics, Harvard Medical School, Boston, MA 02115, United States; Department of Biomedical Informatics, Harvard Medical School, Boston, MA 02115, United States; Department of Biosciences, Nottingham Trent University, Nottingham, NG1 4FQ, United Kingdom; Office of Cyber Infrastructure and Computational Biology, National Institute of Allergy and Infectious Diseases, National Institutes of Health, Bethesda, 20892, United States; Office of Cyber Infrastructure and Computational Biology, National Institute of Allergy and Infectious Diseases, National Institutes of Health, Bethesda, 20892, United States; Centre of Excellence for Biomedical Tuberculosis Research, South African Medical Research Council Centre for Tuberculosis Research, Stellenbosch University, Stellenbosch, Western Cape, 7602, South Africa; Office of Cyber Infrastructure and Computational Biology, National Institute of Allergy and Infectious Diseases, National Institutes of Health, Bethesda, 20892, United States; Division of Infectious Diseases, Chobanian & Avedisian School of Medicine, Boston University, Boston, MA 02118, United States; Centre of Excellence for Biomedical Tuberculosis Research, South African Medical Research Council Centre for Tuberculosis Research, Stellenbosch University, Stellenbosch, Western Cape, 7602, South Africa; Department of Biomedical Informatics, Harvard Medical School, Boston, MA 02115, United States; Department of Data Science, Dana-Farber Cancer Institute, Boston, MA 02215, United States; Broad Institute of Harvard and MIT, Cambridge, MA 02142, United States; Department of Biosciences, Nottingham Trent University, Nottingham, NG1 4FQ, United Kingdom; Unit of Mycobacteriology, Institute of Tropical Medicine, Antwerp, 2000, Belgium; Department of Biomedical Informatics, Harvard Medical School, Boston, MA 02115, United States; Pulmonary and Critical Care Medicine, Massachusetts General Hospital, Boston, MA 02114, United States

## Abstract

**Summary:**

Pan-genome analysis is a fundamental tool for studying bacterial genome evolution; however, the variety in methods used to define and measure the pan-genome poses challenges to the interpretation and reliability of results. Using *Mycobacterium tuberculosis*, a clonally evolving bacterium with a small accessory genome, as a model system, we systematically evaluated sources of variability in pan-genome estimates. Our analysis revealed that differences in assembly type (short-read versus hybrid), annotation pipeline, and pan-genome software, significantly impact predictions of core and accessory genome size. Extending our analysis to two additional bacterial species, *Escherichia coli* and *Staphylococcus aureus*, we observed consistent tool-dependent biases but species-specific patterns in pan-genome variability. Our findings highlight the importance of integrating nucleotide- and protein-level analyses to improve the reliability and reproducibility of pan-genome studies across diverse bacterial populations.

**Availability and implementation:**

Panqc is freely available under an MIT license at https://github.com/maxgmarin/panqc.

## 1 Introduction

Even within the boundaries of defined bacterial species, gene content can vary substantially (Tettelin *et al.* 2005, Medini *et al.* 2020). The concept of the pan-genome, often defined as the union of all genes found across a population (Vernikos 2020), emerged from the necessity to describe this variability in genomic content. Originally developed in the context of bacterial genomics, the pan-genome concept is now applied to genome comparison across the tree of life (Richard 2020). Genes in the pan-genome are typically divided into two categories: core genes, which are shared by nearly all members of a population, and accessory genes, found only in a subset of the population (Vernikos 2020). Multiple mechanisms drive variability in bacterial genomic content, including horizontal gene transfer, recombination, sequence duplication, deletion, and rearrangement (Lassalle and Didelot 2020). With increasing frequency, pan-genome analyses are generating new insights into the genetic diversity and adaptability of bacterial populations, with important implications for fields such as medicine, agriculture, and environmental science (Tettelin *et al.* 2005, Croucher *et al.* 2014, Rouli *et al.* 2015, Pacheco-Moreno *et al.* 2021, Rosconi *et al.* 2022, Yebra *et al.* 2022).

Numerous bioinformatic tools have been developed to analyze genome content within and between species, but they often differ in how they define and measure the pan-genome. For example, a recent review identified over 38 distinct pan-genome analysis pipelines, reflecting the diversity of available tools and approaches (Vernikos 2020). Although a wide variety of tools exist, most pan-genome analyses follow a common structure and depend on two key decisions: (i) the unit of sequence compared across genomes and (ii) the criteria used to assess sequence similarity and homology. For example, analyses may compare the entire genome sequence or instead focus solely on annotated genes. In gene-focused approaches, an additional consideration is whether to compare nucleotide sequences or their predicted amino acid translations. Even after a sequence unit is selected, comparing sequences involves selecting a comparison method (e.g. alignment or k-mer-based approaches) and setting thresholds for the level of similarity required to consider sequences equivalent (Fu *et al.* 2012, Hauser *et al.* 2016, Steinegger and Söding 2018, Li *et al.* 2020). These methodological choices—varying in resolution and sensitivity—can substantially influence both the amount and type of detected variation, shaping downstream interpretation of the pan-genome.

Adding to these methodological complexities, the set of genomes analyzed and whether they adequately capture the population diversity can also influence the predicted pan-genome (Tonkin-Hill *et al.* 2023a). Inclusion of confounding genomes (such as genomes from misidentified species, engineered strains, or contaminants) can distort findings by both reducing core genome estimates and inflating accessory genome estimates (Wu *et al.* 2021). Additionally, the sequencing technology used (e.g. short- versus long-read) and assembly strategy can affect genome assembly accuracy and completeness (Tonkin-Hill *et al.* 2023a). Even small or infrequent errors in a genome sequence may lead to incorrect conclusions about the presence or absence of a gene’s coding sequence (Tonkin-Hill *et al.* 2020, 2023a,b). Together, these sources of bias introduce a further layer of variability that complicates interpretation and hinders comparison across studies.

The bacterial pathogen *Mycobacterium tuberculosis* (*Mtb*) serves as a compelling case study for understanding the challenges of pan-genome analysis. *Mtb* differs from many bacteria for its lack of horizontal gene transfer (HGT) and interstrain recombination (Boritsch *et al.* 2016, Gagneux 2018). This clonal evolution has led to a slowly evolving population with high genome conservation and limited structural variation (Boritsch *et al.* 2014, Gagneux 2018, Orgeur *et al.* 2024). Comparative genomic studies over the past two decades have shown that the *Mtb* accessory genome is shaped primarily by small, lineage-specific deletions and gene disruptions (Brosch *et al.* 2002, Tsolaki *et al.* 2004, Soler-Camargo *et al.* 2022, Behruznia *et al.* 2024, Silva-Pereira *et al.* 2024). A recent analysis evaluating the frequency of gene disruptions across a diverse collection of *Mtb* isolates found that approximately 16% of protein-coding genes were pseudogenized in at least one strain (Soler-Camargo *et al.* 2022). Another notable feature of the *Mtb* genome is the presence of the pe and ppe gene families, which together account for approximately 7% of its coding potential (Gröschel *et al.* 2016, Ates 2020, Gupta and Alland 2021). Certain sub-families (such as PE-PGRS and PPE-MPTR) are particularly prone to frameshift mutations and pseudogenization due to their repetitive sequence structure (Banu *et al.* 2002, Ates 2020). These characteristics make *Mtb* a methodologically challenging case for pan-genome analysis.

Despite substantial evidence of remarkable genome content conservation in *Mtb*, published estimates of its pan-genome size vary greatly, ranging from 506 to 7618 accessory genes (Kavvas *et al.* 2018, Reis and Cunha 2021, Behruznia *et al.* 2024, Silva-Pereira *et al.* 2024). These dramatic differences in results appear to be primarily driven by the methodological choices used in each study. For instance, studies with largest accessory genome estimates typically used tools that define gene absence based solely on CDS amino acid clustering, whereas smaller estimates came from methods that evaluated gene presence/absence at both the CDS and nucleotide level. Additionally, the largest accessory genome predictions came from studies using short-read assemblies, which are prone to fragmentation and assembly errors, whereas the smaller estimates more often relied on complete genomes produced through hybrid assembly. These discrepancies highlight how methodological differences can lead to drastically different pan-genome estimates, even for an organism with no active HGT.

In this work, we focus on understanding the confounding factors and biases in bacterial pan-genome analysis. To achieve this, we used a curated dataset of *Mtb* isolates to systematically benchmark different analysis approaches. This dataset allowed us to examine how assembly quality, annotation pipelines, and pan-genome prediction software affect the results of pan-genome analysis. We first built a pan-genome graph to characterize structural variation between *Mtb* genomes. We found that a majority of the structural variation in the *Mtb* genome involves reconfiguration of existing nucleotide sequence content, instead of loss or gain of novel genomic sequences. Then, we benchmarked common bacterial pan-genome analysis tools and found that several pipelines are prone to overinflating the size of the accessory genome due to CDS annotation discrepancies, and that this pitfall can be worsened by the use of fragmented short-read assemblies as input. Finally, to highlight broader challenges in pan-genome analysis, we expanded our benchmarking to include curated datasets of *Escherichia coli* and *Staphylococcus aureu*s, two phylogenetically distinct pathogens of clinical relevance.

## 2 Materials and methods

### 2.1 Dataset of clinical *Mtb* isolates with long- and short-read WGS

We compiled a dataset of 151 *Mtb* isolates with both short-read (Illumina) and long-read (Oxford Nanopore, PacBio) sequencing data. This includes both previously published data (*n* = 143) and newly sequenced isolates (*n* = 8, PacBio HiFi and Illumina WGS). Due to significant variations in sequencing depth and read lengths of generated long-read WGS, we used stringent selection criteria for inclusion in analysis. Specifically, we selected only isolates that could be assembled into a single, circular contig when using the Flye long-read genome assembler. This selection was crucial to ensure that the hybrid assemblies reflect truly complete *Mtb* genomes. Supplementary File S2 details all relevant ENA/SRA run accessions and metadata for all *Mtb* genome sequencing data used.

### 2.2 H37Rv reference genome and annotations

The H37Rv (NCBI Accession: NC_000962.3) genome sequence and annotations was used as the standard reference genome for all analyses involving *Mtb*. Functional category annotations for all genes of H37Rv were downloaded from Release 3 of MycoBrowser (https://mycobrowser.epfl.ch/releases). The H37Rv reference sequence was also annotated with the Bakta (v4.8) and PGAP (v6.4) pipelines for comparison with the official H37Rv annotations. The DNA Features Viewer python library was used to generate programmatic visualizations of the NCBI, PGAP, and Bakta H37Rv annotations (Zulkower and Rosser 2020) shown in Supplementary Fig. S5 and in Supplementary Files S9 and S10.

### 2.3 Selection of a diverse dataset of *E. coli* genome assemblies

A subset of 50 published genomes were selected from Shaw *et al.* a previous analysis of *E. coli* genomic diversity (Shaw *et al.* 2021). In this study, all genomes were assembled using an hybrid approach using both long and short-read genome sequencing data. In order to assure a diverse set of genomes, representative subset of isolates from the following nine *E. coli* phylotypes were selected from published metadata: A, B1, B2, C, D, E, F, G, and clade V. To complement the available published hybrid assemblies, the paired-end short-read genome sequencing data for each isolate was downloaded from the NCBI Sequence Read Archive for *de novo* short-read assembly. Metadata for all evaluated *E. coli* isolates, including assembly and sequencing run accessions, are provided in Supplementary File S2.

### 2.4 Selection of a diverse dataset of *S. aureus* genome assemblies

A dataset of 68 *S. aureus* genomes were selected from a published study of pan-genome variation of *S. aureus* clinical isolates (Houtak *et al.* 2023). In this study, all published genomes were assembled using a hybrid approach combining both long and short-read genome sequencing data. To complement the existing published hybrid assemblies, the paired-end short-read genome sequencing data for each isolate was obtained from the NCBI Sequence Read Archive for *de novo* short-read assembly.

### 2.5 Hybrid genome assembly with long and short read sequencing

The hybrid genome assembly and polishing process was tailored to the specific requirements of various long-read WGS platform and chemistry versions used for analysis (PacBio subreads [RSII and Sequel II], ONT v9.4.1, PacBio CCS/HiFi [Sequel II] reads), as well as taking into account the software versions available at the time of data processing. Refer to the Supplementary Methods for the exact combination of softwares and parameters used for genome assembly.

### 2.6 Short read *de novo* genome assembly

The following assembly approach was applied to all paired-end Illumina WGS data from *Mtb*, *E. coli, and S. aureus* isolates. First, the paired-end reads were trimmed with Trimmomatic (v0.39) (Bolger *et al.* 2014). After read processing, *de novo* short-read assemblies were then generated using Unicycler (v0.4.8), which serves as an assembly optimizer for SPAdes (v3.13) (Wick *et al.* 2017, Prjibelski *et al.* 2020). Prior to assembly of the *Mtb* isolates, the trimmed reads were additionally filtered using Kraken2 to keep only reads that were confidently classified as *Mtb* complex (MTBC, TaxID: 77643) (Wood *et al.* 2019). After assembly of the *Mtb* isolates, Kraken2 was used to select only contigs that were classified as MTBC (TaxID: 77643). This Kraken2 filtering was performed to minimize chances of contaminating contigs from other species being included in the pan-genome analysis using short-reads. The standard complete Kraken2 RefSeq database was used for all sequence classification.

### 2.7 Phylogeny inference of *Mtb* dataset

Genetic variants relative to the H37Rv reference genome were inferred for each hybrid genome assembly using minimap2 and paftools.js (Li 2018). A concatenated SNP alignment was then generated by identifying and extracting single nucleotide polymorphisms (SNPs) from each genome assembly using bcftools (Danecek *et al.* 2021). From the SNP alignment, a maximum likelihood phylogeny was inferred using IQ-Tree with the general time reversible model and a SNP ascertainment bias correction (Minh *et al.* 2020).

### 2.8 Phylogeny inference of *E. coli* and *S. aureus* datasets

To generate a core genome alignment for the *E. coli* and *S. aureus* datasets respectively, the hybrid assembly genomes for each dataset were processing using Panaroo with the following settings: --merge_paralogs, --clean-mode strict, --remove-invalid-genes, --alignment core, --aligner mafft. From the core gene alignment FASTA, a maximum likelihood phylogeny was inferred using IQ-Tree with the general time reversible model (Minh *et al.* 2020).

### 2.9 Assessment of high-level genome sequence similarity

For the *Mtb* (*n* = 151), *E. coli* (*n* = 50), and *S. aureus* (*n = *68) datasets, FastANI version (v1.3) run with default parameters to estimate Average Nucleotide Identity (ANI) between all pairs of complete genomes (Jain *et al.* 2018). SourMash version (v4.8.2) was used to calculate the Jaccard Similarity of all unique 31 bp k-mers between each pair of complete genomes within a dataset (Pierce *et al.* 2019). To calculate the profile of all canonical 31 bp k-mers for each genome, the sourmash sketch dna command was run with the -p scaled = 1 parameter. The -p scaled = 1 parameter forces the comparison of the complete k-mer set (no downsampling) of each genome. All k-mer signatures were then input into the sourmash compare command with default parameters. The Seaborn library was used to visualize heatmaps of estimated ANI and k-mer Jaccard Similarity across each bacterial population (Waskom 2021).

### 2.10 Construction of the *Mtb* SV pan-genome graph

The *Mtb* SV pan-genome graph was built with Minigraph (v0.19, default parameters) using H37Rv as the initial reference and with all 151 complete genome assemblies as input (Li *et al.* 2020). GFAtools was used for all graph manipulations and reformatting of bubble region and node information. The Bandage software was used for visualization of the resulting *Mtb* SV pan-genome graph (Wick *et al.* 2015).

### 2.11 Genome annotation

All hybrid and short-read assemblies were annotated with the Bakta (v4.8) and PGAP (v6.4) annotation pipelines (Tatusova *et al.* 2016, Schwengers *et al.* 2021). The GFF annotation files output by each annotation pipeline were used as input to all pan-genome analysis pipelines evaluated. All genome assemblies (Hybrid and Short-read) and their respective annotations used in this study are available on Zenodo (10.5281/zenodo.10846276).

### 2.12 Benchmarking pan-genome analysis pipelines

We benchmarked four gene-centric pan-genome analysis pipelines (Panaroo, Roary, PPanGGolin, and Pangene) across datasets from three bacterial species: *Mtb*, *E. coli*, and *S. aureus*. All of these analyses followed a standardized three-step workflow. First, genome assemblies were selected for analysis, using either hybrid assemblies (based on long- and short-read sequencing) or short-read-only *de novo* assemblies. Second, each genome was annotated *de novo* using Bakta or PGAP, producing gene and coding sequence (CDS) annotations (Tatusova *et al.* 2016, Schwengers *et al.* 2021). Third, annotated assemblies were analyzed using each pan-genome analysis software with varying internal parameter settings to assess the influence of gene clustering thresholds and heuristics on pan-genome estimates. For *Mtb*, analyses were conducted using both Bakta- and PGAP-annotated assemblies. For *Eco* and *Sau*, only Bakta annotations were used to simplify cross-species comparisons.

Each pan-genome tool was run across a range of parameter combinations to capture the impact of different gene clustering setterings. For Panaroo, we varied the accessory gene filtering stringency using the --clean-mode parameter (strict, moderate, or sensitive) and toggled --merge_paralogs to control whether paralogs were merged or retained as separate genes. For Roary, we varied the minimum amino acid identity threshold used to cluster protein sequences (-i set to 80, 90, or 95) and whether paralogous sequences were merged (-s flag enabled or not). PPanGGolin was evaluated using nine combinations of minimum alignment coverage (--coverage set to 0.8, 0.9, or 0.98) and minimum sequence identity thresholds (--identity set to 0.6, 0.8, or 0.9). For Pangene, we followed its standard three-step workflow. First, we used CD-HIT to cluster CDSs based on amino acid similarity, varying the clustering threshold (-c) across 0.90, 0.95, and 0.98. Second, the representative protein sequence from each CDS cluster was aligned to analyzed genomes using Miniprot (protein-to-genome alignment), varying the alignment identity threshold (--outs) across 0.90, 0.95, 0.98, and 0.99 (Li 2023). Third, the resulting Miniprot alignments were processed by Pangene to build a gene-level pan-genome graph and generate a gene presence/absence matrix.

From these results we then evaluated the number of core genes (present in ≥99% of assemblies), and the number of accessory genes (present in <99% of assemblies) defined by the gene presence/absence matrix of each analysis output. A complete summary of all pan-genome estimates generated for this study can be found in Supplementary File S8.

### 2.13 Overview of the panqc pipeline

The panqc nucleotide redundancy correction pipeline adjusts for both CDS annotation discrepancies and nucleotide redundancy within an estimated pan-genome with two steps. In step one, all genes absent at the CDS level are aligned to each corresponding assembly at the nucleotide level. This step is implemented using minimap2 to align the nucleotide sequence of each gene to the corresponding genome assembly (Li 2018). By default if the absent CDS’s gene sequence is found with both 90% coverage and sequence identity it will be marked as a CDS annotation discrepancy, meaning the gene is absent at CDS level but present at the nucleotide level. The align coverage and identity thresholds are usable definable parameters. Next, all genes are re-clustered and merged using a nucleotide k-mer based metric of nucleotide similarity. Cases where two or more genes are divergent at the protein level but highly similar at the nucleotide level will be merged into a single “nucleotide similarity gene cluster.” An adjusted gene presence/absence matrix is then produced such that all gene clusters in the input are merged if they share substantial DNA sequence similarity.

In the DNA k-mer similarity graph used for reclustering of sequences, distances between genes are specifically computed as the maximum Jaccard Containment between their k-mer sets. The Jaccard Containment between the k-mer sets of genes A and B is calculated as Jaccard Containment(A, B)=|A ∩ B| ⁄|A|, where |A| denotes the total number of k-mer in the set A, and |A ∩ B| represents the number of k-mers shared between A and B. To account for different gene lengths, we use the maximum Jaccard Containment as this ensures that the k-mer similarity will be set to 1 if a shorter gene’s k-mer set is fully contained within a longer gene’s set.

## 3 Results

### 3.1 Curating a dataset of high quality *Mtb* genomes

**Figure 1.**
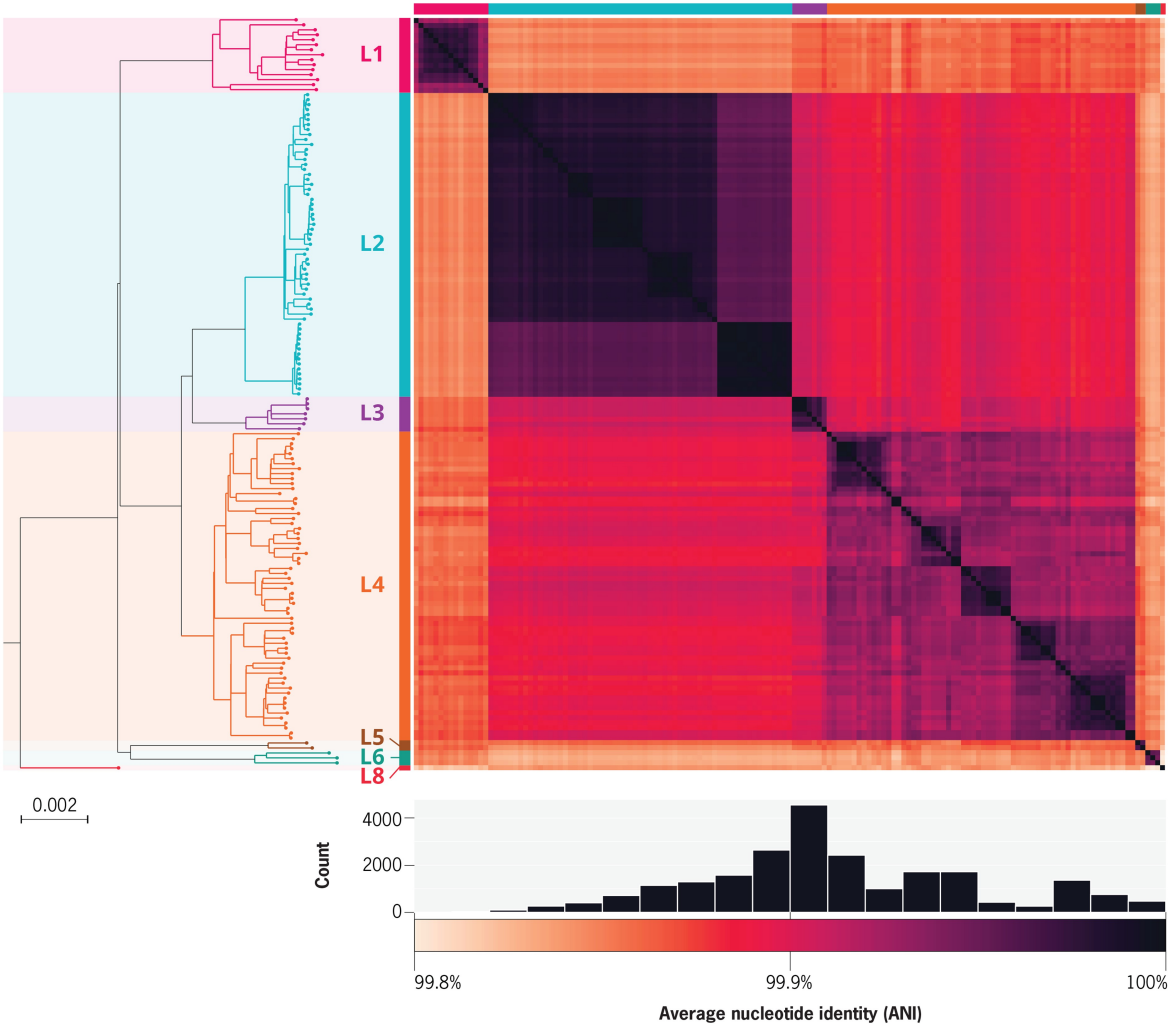
Summary of dataset of 151 complete *Mtb* genomes. Left: Maximum likelihood phylogeny of all 151 genomes, colored according to their lineage (L1-6, L8). Right: Heatmap of pairwise ANI. Below: Distribution of pairwise ANI values, and corresponding heatmap colorbar.

We curated a collection of 151 complete assemblies of *Mtb* derived from diverse human adapted isolates. This was done using six previously published collections, as well as eight isolates newly sequenced for this study (Chiner-Oms *et al.* 2019, Lee *et al.* 2020, Ngabonziza *et al.* 2020, Peker *et al.* 2021, Marin *et al.* 2022, Hall *et al.* 2023). Each isolate was sequenced using both short- and long-read technologies (Oxford Nanopore and PacBio), and both a short-read (SR) *de novo* assembly and a hybrid genome assembly (long-read *de novo* assembly with short-read polishing) were generated. The resulting dataset includes *Mtb* lineages 1–6 and 8, spanning the global diversity of the *Mtb* phylogeny (Fig. 1). The curated genomes exhibit high sequence similarity and conserved genome characteristics: 99.8%–100% pairwise average nucleotide identity (ANI), 0.94–0.99 pairwise k-mer jaccard similarity, a genome size of 4.38–4.44 Mb, 4020–4135 predicted proteins (CDSs), and 65.6%—65.6% GC content (Fig. 1 and Supplementary Fig. S2, Supplementary Table S1). As expected, short-read assemblies were consistently more fragmented, had a lower cumulative length, and fewer predicted coding sequences compared to their hybrid counterparts.

### 3.2 Most structural variation in *Mtb* is attributed to rearrangements of existing sequence, rather than to novel sequence content

We next aimed to characterize the structural variation (SV) landscape across our dataset of complete *Mtb* genomes and quantify its contribution to accessory genome content across the population. This analysis focused on distinguishing between SVs that generate novel accessory sequence from those involving the rearrangement or duplication of existing genomic material. To achieve this, we constructed a pan-genome graph of SVs, using the Minigraph algorithm (Li *et al.* 2020). The Minigraph algorithm identifies all SVs ≥50 bp by iteratively aligning genomes and incorporating new variants into a graph. By design, this approach prevents the collapse of repeated sequences, preserving genomic context and co-linearity of the identified SVs.

**Figure 2.**
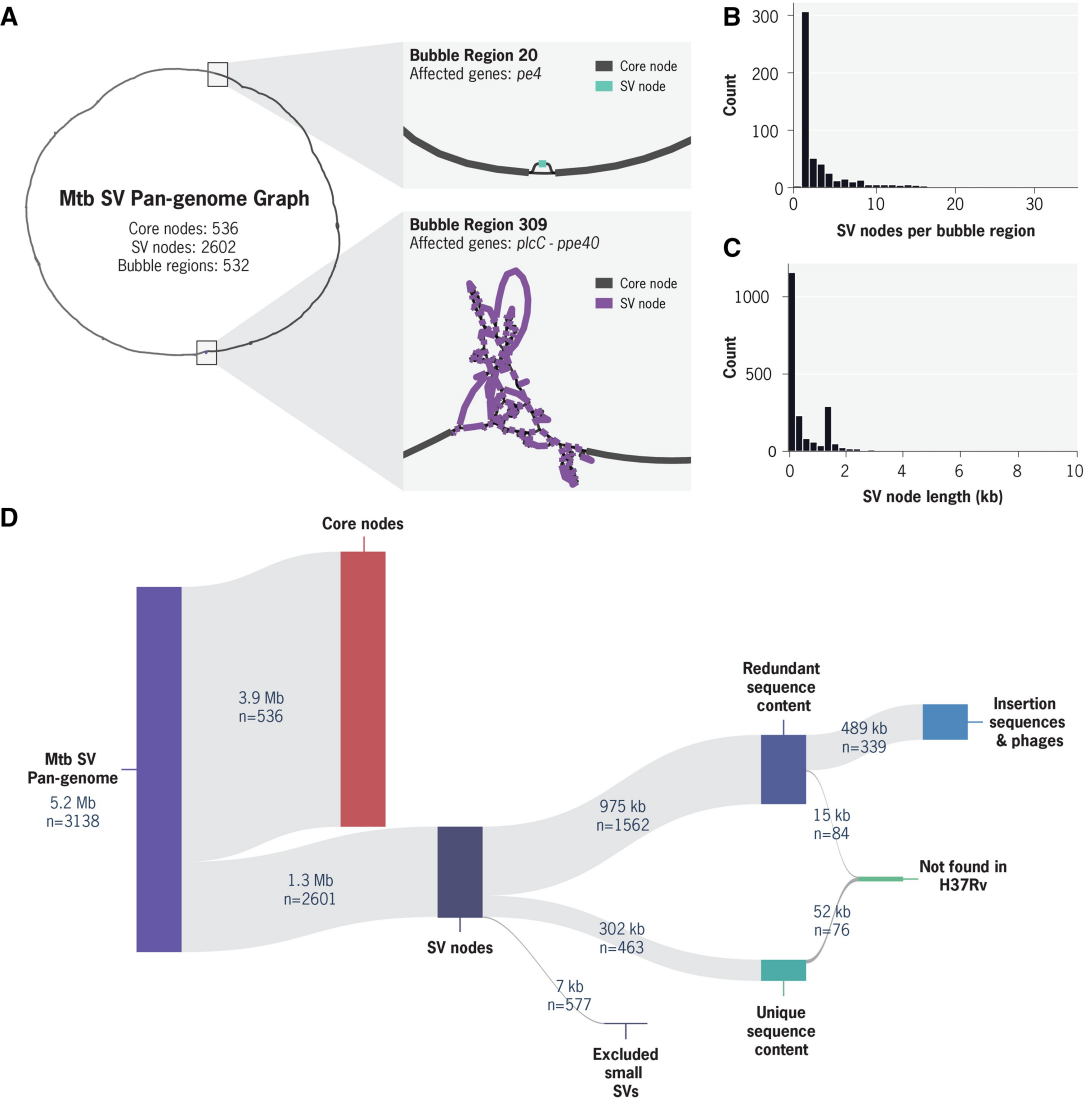
Characteristics of *Mtb* SV pan-genome graph. (A) Left: Circle representing the high-level view of the *Mtb* SV pan-genome graph. Right: Two bubble regions shown in detail. Bubble Region 20 is representative of regions with a simple insertion/deletion, containing a single SV node (186 bp) in gene pe4 (Rv0160c). Bubble region 309 is representative of a complex bubble region, containing 88 SV nodes (55 759 bp) spanning from gene plcC (Rv2349c) to ppe40 (Rv2356c). (B) Distribution of the number of SV nodes per bubble region. (C) Distribution of SV node length. (D) Hierarchical breakdown of Core and SV nodes in specific categories of interest, showing number of nodes and cumulative length.

We next classified the nodes of the pan-genome graph into Core nodes (genomic regions present across all isolates), and SV nodes (representing structural variants found between genomes). The pan-genome graph contained 536 Core nodes, with a cumulative length of 3.9 Mb, and 2602 SV nodes, with a cumulative length of 1.3 Mb (Fig. 2). A genomic region containing structural variation may consist of multiple connected SV nodes; such regions are referred to as bubble regions in this study. They can range from simple insertions or deletions to highly complex regions with multiple distinct rearrangements. Two representative examples affecting genes belonging to the *pe* and *ppe* gene families are shown in Fig. 2A. In total, there were 535 distinct bubble regions identified.

Given the large number of SV nodes detected, we aimed to understand which nodes represented truly novel sequence content and those involving the rearrangement of existing sequences. For this, we implemented a computationally efficient k-mer comparison method that classified nodes as unique or redundant by assessing shared k-mer content across all nodes in the graph (Section 2). We identified 463 SV nodes with unique k-mer content (Fig. 2D), indicating that only 23% (302 kb) of the total cumulative length of SV nodes represent novel sequence content. These results establish a robust baseline for the expected amount of accessory genome content in downstream analyses.

We next classified redundant SV nodes according to the *Mtb* gene categories they comprise (Supplementary Table S2). Notably, we found that more than half (339 SV nodes, 489 kb of cumulative length) belonged to the Insertion sequences and phages category (Fig. 2D). Upon further inspection, a single type of insertion element, IS6110, was responsible for the vast majority (455 kb) of the redundant SV nodes. We found that only a minor fraction of the SV nodes (67 kb, 5% of the total length), represent sequences completely absent from the H37Rv reference genome. These SV nodes were spread across 65 bubble regions in the graph, and contain known deletions unique to specific *Mtb* lineages, such as TbD1 (Brosch *et al.* 2002, Bottai *et al.* 2020) (Supplementary Fig. S3). The 18 largest bubble regions with sequence absent from H37Rv are highlighted in Supplementary Table S3.

### 3.3 The choice of software and specific pipeline parameters can substantially impact pan-genome size estimates

In bacterial pan-genome analysis, most workflows begin with *de novo* genome annotation, followed by homology-based clustering of annotated coding sequences (CDS), but after this initial step, pipelines can diverge substantially in how they adjust and refine these clusters. Because some published studies appear to overestimate *Mtb* accessory genome size, we assessed variability in *Mtb* pan-genome estimates when using four commonly used pipelines: Panaroo, Roary, PPanGGolin, and Pangene (Page *et al.* 2015, Gautreau *et al.* 2020, Tonkin-Hill *et al.* 2020, Li *et al.* 2024). We investigated three key parameter types known to affect analysis outcomes: (i) the assembly type of the input genomes (*de novo* short-read assembly versus hybrid genome assembly), (ii) the gene annotation pipeline applied to these genomes (Bakta or PGAP), and (iii) the gene clustering parameters of the pan-genome software (sequence identity threshold, merging of paralogs, and pipeline heuristics).

**Figure 3.**
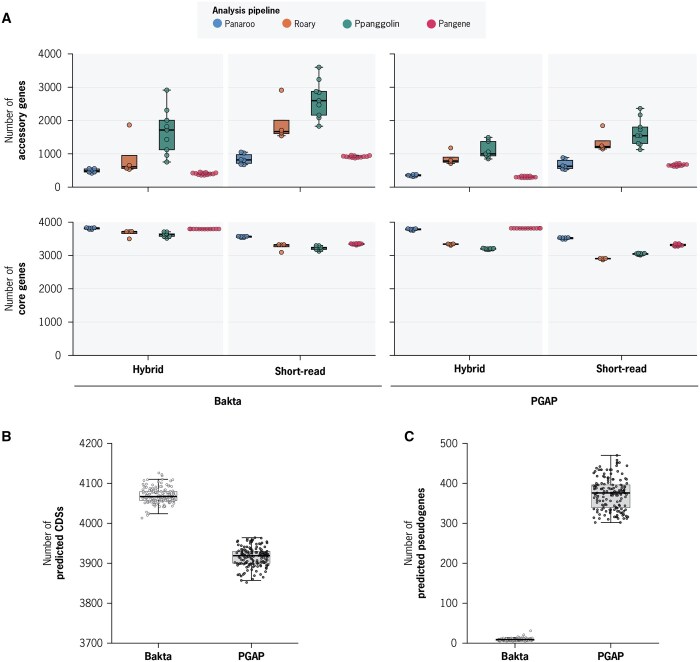
Comparison of *Mtb* pan-genome predictions across different analysis parameters. (A) Comparison of the number of core and accessory genes estimated for the identical population of 151 *Mtb* isolates across all tested parameters: Assembly type (hybrid versus short-read), annotation pipeline (Bakta versus PGAP), and pan-genome software (Panaroo, Roary, PPanGGolin, and Pangene). Each data point represents a different set of gene clustering parameters of the specific software. (B) Number of predicted CDS features annotated by Bakta and PGAP across all hybrid *Mtb* genomes. (C) Number of predicted pseudogene features annotated by Bakta and PGAP across all hybrid *Mtb* genomes.

Despite analyzing an identical population of 151 *Mtb* isolates, results across parameter combinations varied widely, with accessory gene estimates ranging from 277–3602 and core genes from 2868–3833 (Fig. 3A and Supplementary Fig. S4). We detected distinct trends in how different parameters influenced the results. First, using short-read assemblies systematically resulted in smaller core genome, and larger accessory genome estimates. Using short-read assemblies predicted on average 551 more accessory genes and 368 less core genes, compared to using their respective hybrid genome assemblies. We found that when using short-read assemblies, 7%–13% of all predicted gene absences were a direct consequence of assembly failures (Supplementary Results, Supplementary Table S6).

Additionally, the annotation pipeline used also had a substantial impact on analysis results. Compared to PGAP, using Bakta annotations consistently produced larger pan-genome size estimates, with an average increase of 430 total genes (Fig. 3A). We investigated the differences between the two annotation pipelines and found that PGAP consistently annotated fewer CDSs and more pseudogenes per genome (Fig. 3B and C, Supplementary Fig. S5, Supplementary Tables S3 and S4, Supplementary Results). This effect was substantial: PGAP annotated between 302–470 pseudogenes per genome, whereas Bakta annotated only 5–31. Notably, this corresponds to 7%–11% of genes being annotated as pseudogenes in PGAP, which is critical given that pseudogenes are excluded from the initial CDS clustering steps in all evaluated pan-genome pipelines.

Finally, we found that each software tool differed in how consistent the results were under varying input parameters. Panaroo and Pangene were the most consistent across all tested variables, producing accessory genome size estimates that ranged from 313–1050 and 277–959 genes, respectively. In contrast, Roary and PPanGGolin accessory genome size estimates ranged from 538–2912 and 755–3602 genes, respectively (Fig. 3A). The robustness of Panaroo and Pangene suggests that these tools are less sensitive to discrepancies in CDS annotations, likely due to their cluster-refinement strategies, which incorporate nucleotide-level information rather than relying primarily on CDS annotations. As a result, they are more reliable when handling data of varying quality or when identifying complete gene gain or loss at the nucleotide level.

### 3.4 In *Mtb*, accessory genome inflation is driven by differences in coding sequence annotations rather than by actual nucleotide content variation

Given the drastic variability observed in *Mtb* pan-genome results under different parameters, we aimed to identify which trends were generalizable to other bacterial species and which were specific to *Mtb’*s unique genomic features. As with the *Mtb* dataset, we curated two additional datasets consisting of 50 *Escherichia coli* (*Eco*) and 68 *Staphylococcus aureus* (*Sau*) isolates, each sequenced using both short- and long-read technologies (Shaw *et al.* 2021, Houtak *et al.* 2023). (Supplementary Figs S7 and S8). These bacterial species were selected because they belong to phylogenetically distant groups, and, unlike *Mtb*, exhibit horizontal gene transfer and substantially greater sequence divergence within their populations. While the pairwise ANI within *Mtb* ranged from 99.8%–100%, it ranged from 90.6%–100% and 97.2%–100%, in the *Eco* and *Sau* datasets respectively (Supplementary Figs S7 and S8).

**Figure 4.**
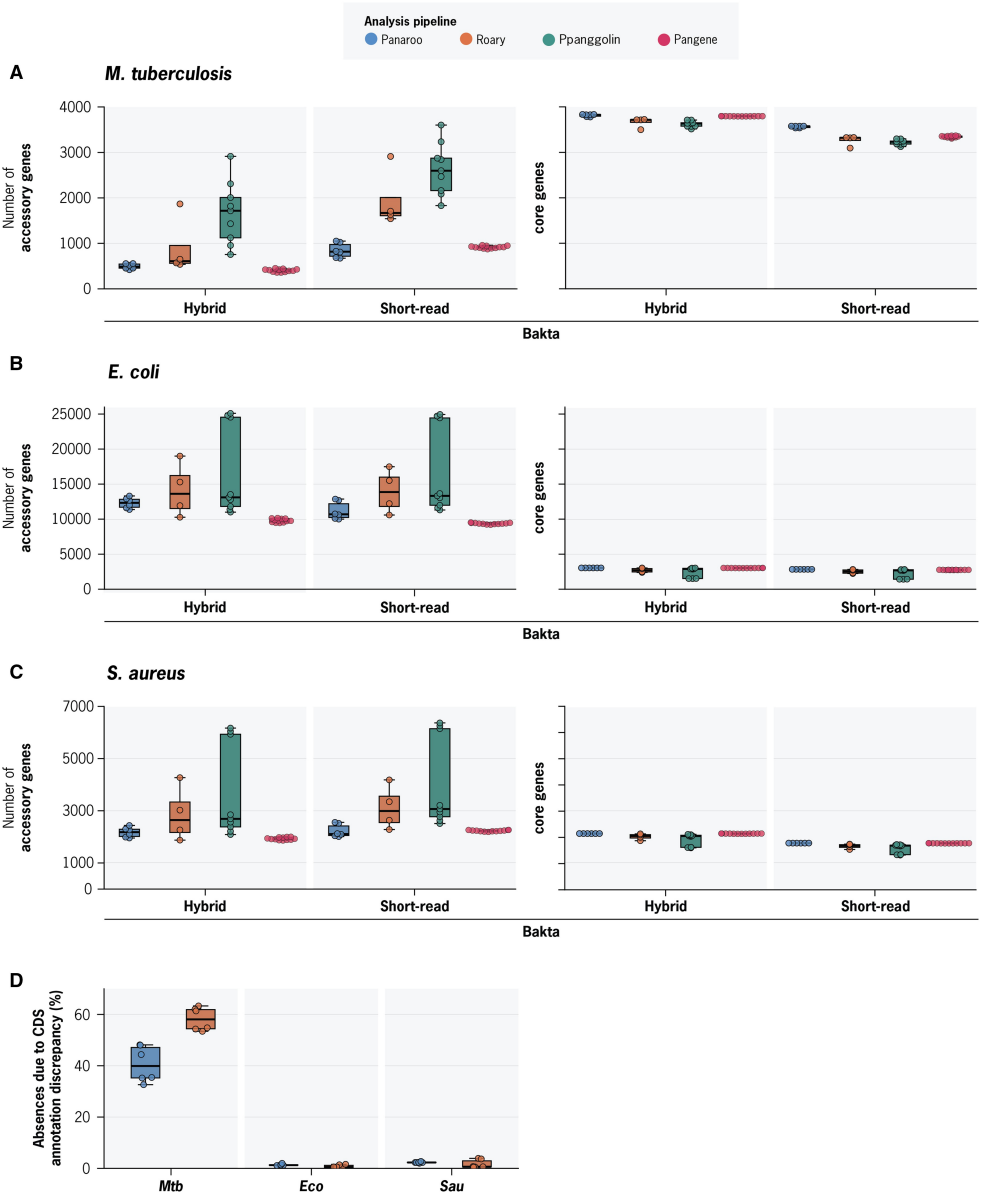
Pan-genome tool comparison across three different bacterial species (A–C) Core and accessory genome estimates for *Mtb*, *Eco*, and *Sau* datasets across all tested parameters: Assembly type (hybrid versus short-read), and pan-genome software (Panaroo, Roary, PPanGGolin, and Pangene). Each data point represents a different set of gene clustering parameters of the specific software. (D) Percentage of gene absences due to CDS annotation discrepancy across *Mtb*, *Eco*, *Sau*. Each data point represents a different set of gene clustering parameters for Panaroo or Roary.

We conducted the same benchmarking experiments using Panaroo, Roary, PPanGGolin, and Pangene, using short-read and hybrid assemblies annotated with Bakta. Consistent with our findings in *Mtb*, the pan-genome estimates in *Eco* and *Sau* were highly sensitive to the specific parameters evaluated. Pan-genome estimates for the *Eco* population ranged from 1418–3036 core genes and 9201–25 101 accessory genes, while pan-genome estimates for the *Sau* population ranged from 1323–2146 core genes and 1862–6366 accessory genes (Fig. 4A–C). Likewise, as observed with *Mtb*, Panaroo and Pangene deliver more consistent results, and exhibit greater robustness to parameter selection. However, in contrast with our findings in *Mtb*, using short-read assemblies had an opposite effect on overall pan-genome size in *Eco* and *Sau*. For *Mtb*, using short-read assemblies on average increased the pan-genome size by 278 genes while for *Eco* and *Sau* it resulted in an average decrease of 559 and 99 total genes respectively.

For all three species, we aimed to understand to what extent discrepancies in pan-genome estimates could be attributed to differences in CDS annotation. Variations in predicted amino acid sequences can arise from several sources, including assembly errors, frameshift mutations, or other genuine mutations that disrupt coding sequences. Yet, when the underlying nucleotide sequence is largely unchanged, such differences likely do not reflect true gene gain or loss. We found that, on average, 49% of all genes predicted to be absent *Mtb* pan-genome predictions were caused by discrepancies in CDS annotation (Fig. 4D). In contrast to *Mtb*, *Eco*, and *Sau* had a minimal proportion (∼1%) of gene absences caused by CDS annotation discrepancies (Fig. 4D, Supplementary Tables S6–S8). These results highlight a challenge unique to *Mtb*, in which repetitive sequences, frameshift mutations, and gene pseudogenization can frequently result in CDS annotation discrepancies, which in turn inflates accessory genome size estimation.

### 3.5 Developing a tool to account for nucleotide redundancy within CDS based pan-genome estimates

**Figure 5.**
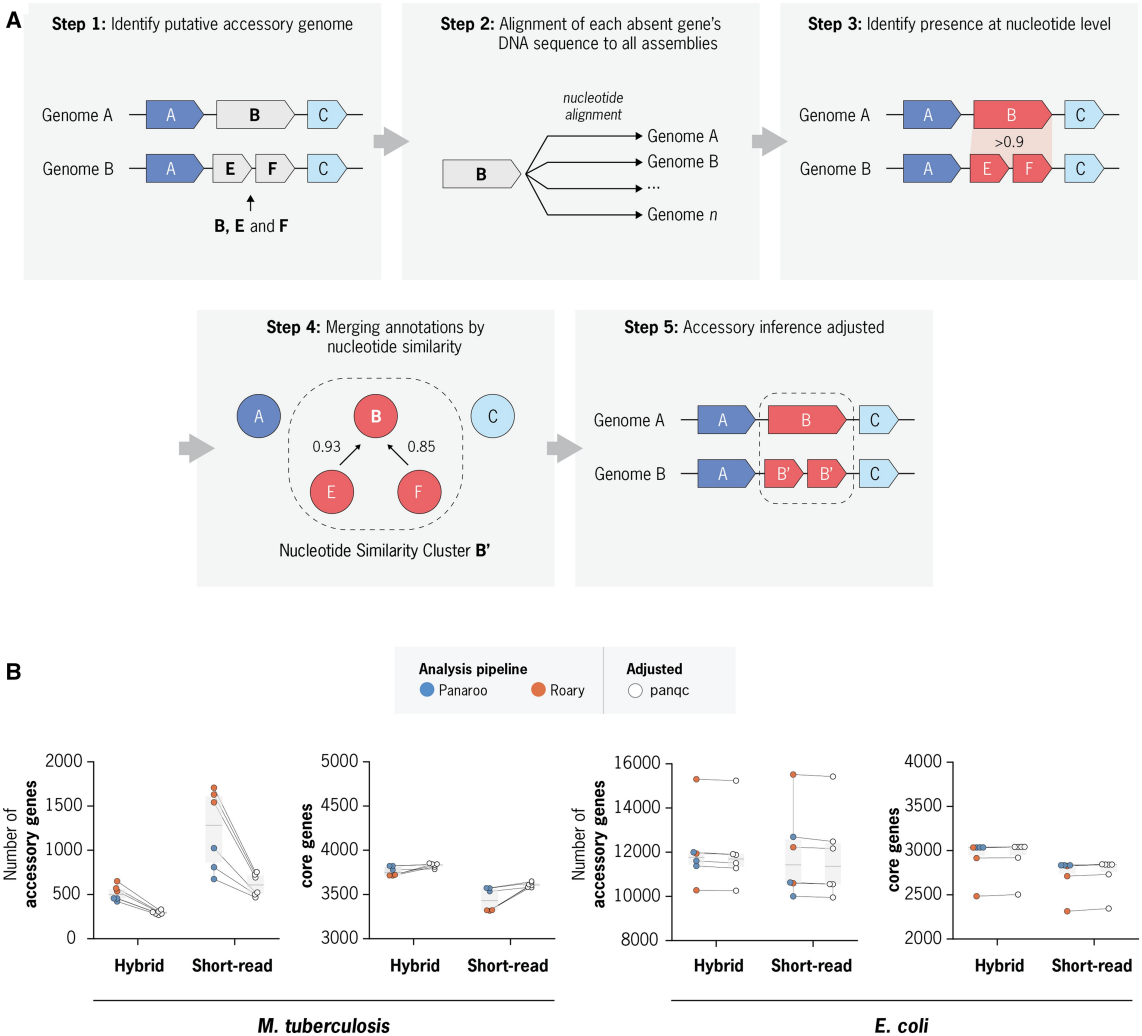
Overview of the panqc nucleotide correction pipeline and panqc adjustment of *Mtb* and *Eco* pan-genome estimates. (A) Diagram of the panqc algorithm: In Step1, all predicted gene absences making up the predicted accessory genome are identified. In Step 2, each absent gene’s nucleotide sequence is aligned against all genomes. In Step 3, alignments are analyzed to identify if the nucleotide sequence is still present despite the previously predicted absence. In Step 4, all genes are clustered based on the similarity of their nucleotide sequences. In Step 5, pan-genome estimates are readjusted accounting for presence/absence of nucleotide sequence. (B) Comparison of Panaroo and Roary pan-genome predictions before and after panqc re-adjustment with default parameters for *Mtb* and *Eco* datasets, for both hybrid and short-read assemblies. Each data point represents a different set of gene clustering parameters for Panaroo or Roary before or after panqc adjustment.

Motivated by our observation that CDS annotation discrepancies can inflate the estimated pan-genome size, we developed panqc. panqc is a software that takes output files from commonly used pan-genome prediction softwares, and readjusts the pan-genome estimates by reclustering CDSs with highly similar nucleotide sequence content. Our algorithm consists of two steps: First, it takes all the CDSs predicted to be absent in a genome, and queries the nucleotide sequence against the associated genome assembly. If the nucleotide sequence is found, with a coverage and sequence identity >90%, the gene is classified as being present at the DNA level, but absent at the CDS level. Second, genes are merged into nucleotide similarity clusters using a k-mer based similarity metric (Fig. 5A, Section 2). The final output is an updated pan-genome estimate that prioritizes differences in nucleotide sequence content over coding sequence differences.

We evaluated the effect of panqc readjustment on compatible pan-genome outputs of Roary and Panaroo for our *Mtb* and *Eco* datasets (Fig. 5A and Supplementary Fig. S9, Supplementary Table S9). Across all *Mtb* estimates, panqc reduced the overall accessory genome size by 420 genes (44%) on average. Even when applied to the most conservative estimate produced by Panaroo (using hybrid assemblies, and the --clean-mode strict and --merge_paralogs options), the pan-genome size was reduced by 139 genes. For *Eco*, panqc modestly reduced the estimated accessory genome size by 80 genes (0.7%) on average (Fig. 5B and Supplementary Fig. S10, Supplementary Table S10). Although the absolute number of genes re-clustered is similar to that of *Mtb*, this represents a much smaller proportion of the overall *Eco* accessory genome (estimated to be 9201–25 101 genes).

## 4 Discussion

In this work, we systematically evaluated how various input data and software parameters influence pan-genome analysis, highlighting critical pitfalls in the process. We benchmarked commonly used bacterial pan-genome prediction softwares: Panaroo, Roary, PPanGGolin, and Pangene. We varied software specific parameters that affect gene clustering heuristics, as well as key characteristics of the input genomes, including assembly type and gene annotation pipeline used. We applied this benchmarking framework to three datasets of phylogenetically distinct bacterial species: *Mtb*, *E. coli*, and *S. aureus*. Across all three species, these parameters had a large impact on pan-genome size estimates, with differences in core and accessory gene counts reaching into the thousands. The extreme variability observed across these results underscores how challenging it is to compare and interpret pan-genome results across studies. Additionally, it emphasizes that researchers must understand and report the underlying assumptions and parameters of the pan-genome analysis tools used.

Although pan-genome results were highly sensitive to parameter choices in all three species, *Mtb* was especially affected by discrepancies in CDS annotations across isolates. This sensitivity is largely due to its high content of repetitive sequences and a high rate of gene pseudogenization (due to frameshift, insertion and deletion mutations). Combined with a genuinely small accessory genome, these factors lead to a disproportionate inflation of accessory gene counts relative to the amount of accessory nucleotide sequence. Supporting this, our graph-based pan-genome analysis revealed that while structural variation exists in *Mtb* genomes, it primarily involves rearrangement of existing sequence, instead of novel nucleotide content. Despite some studies reporting unexpectedly large accessory genomes for *Mtb* (contradicting its well-established genomic conservation) our results suggest that how different pan-genome softwares handle protein coding differences (causing CDS annotation discrepancies) are the main drivers of inconsistency in accessory genome size. These findings raise the intriguing possibility that *Mtb* may compensate for its lack of horizontal gene transfer by leveraging disruptive mutations in coding regions as a major source of adaptive variation.

To address the potential ambiguity that arises from CDS annotation discrepancies, we developed panqc. It takes output files from commonly used pan-genome softwares and allows the user to readjust the pan-genome estimates by reclustering CDSs with highly similar nucleotide sequence content, with transparent controls over how nucleotide redundancy is accounted for. By reporting whether gene presence or absence is due to differences in CDS annotation or nucleotide-level absence, panqc provides valuable context for interpreting pan-genome results. This, in turn, enables more meaningful comparisons between outputs from different tools and parameter choices. We envision that panqc can be used in conjunction with other tools available for quality control of pan-genome estimates, such as Panaroo’s suite of post-processing scripts or Panstripe (Tonkin-Hill *et al.* 2020, 2023b).

While certain research questions can be addressed by focusing primarily on either protein-level or nucleotide-level differences, a comprehensive view of the evolutionary dynamics influencing genome variation will require methods that smartly integrate both levels of sequence information. We anticipate that improvements in the next wave of pan-genome analysis methods will continue to come from approaches that innovate on the integration of nucleotide and amino acid level information in biological meaningful ways.

## Supplementary Material

btaf219_Supplementary_Data

## Data Availability

All SRA/ENA run accessions and associated metadata for all genomic data used in this study can be found in Supplementary File S2. Code for data processing and analysis is available from the following GitHub repository, https://github.com/farhat-lab/mtb-pg-benchmarking-2024paper/. The Snakemake workflow engine was used for data processing(Köster and Rahmann 2012). The panqc software is available in the following GitHub repository, https://github.com/maxgmarin/panqc.
