# Supplementary material for: Pitfalls of bacterial pan-genome analysis approaches: a case study of Mycobacterium tuberculosis and two less clonal bacterial species
Source: Bioinformatics. 2025 May 8;41(5):btaf219. doi: 10.1093/bioinformatics/btaf219 (PMC12119186; doi:10.1093/bioinformatics/btaf219)
Supplement: btaf219_Supplementary_Data [file btaf219_supplementary_data.zip › SF1.SupplementalMaterial.pdf]

Supporting Information for

Pitfalls of bacterial pan-genome analysis approaches: a case study of M. tuberculosis and two less clonal bacterial species

Maximillian G. Marin[1], Natalia Quinones-Olvera[1], Christoph Wippel[1], Mahboobeh Behruznia[2],Michael Harris[3], Brendan M. Jeffrey[3], Brendon Mann[4], Alex Rosenthal[3], Karen R. Jacobson[5], Robin M. Warren[4], Heng Li[1], Conor J. Meehan[2,6], Maha Reda Farhat[1,7*]*

1 Department of Biomedical Informatics, Harvard Medical School, Boston, USA
2 Department of Biosciences, Nottingham Trent University, Nottingham, UK
3 Office of Cyber Infrastructure and Computational Biology, National Institute of Allergy and Infectious Diseases, National Institutes of Health, Bethesda, USA
4 Centre of Excellence for Biomedical Tuberculosis Research, South African Medical Research Council Centre for Tuberculosis Research, Stellenbosch University, Cape Town, South Africa
5 Division of Infectious Diseases, Boston University Chobanian & Avedisian School of Medicine, Boston Medical Center, Boston, USA
6 Unit of Mycobacteriology, Institute of Tropical Medicine, Antwerp, Belgium
7 Pulmonary and Critical Care Medicine, Massachusetts General Hospital, Boston, USA
*Corresponding authors: Maha Reda Farhat, Maximilian Gabriel Marin

To whom correspondence may be addressed.
Email: Maha_Farhat@hms.harvard.edu or maximillian_marin@hms.harvard.edu

This PDF file includes:

Summary of supplemental files
Supplemental Text (Methods & Results)
Supplemental References
Figures S1 to S10
Tables S1 to S12

Summary of supplemental files

File S1. Supplemental Text, Figures, and Tables

File S2. SRA/ENA genome assembly and sequencing accessions for long-read and short-read sequencing used in this study

File S3. Summary of *Mtb* isolate information and genome assembly (Short & hybrid) characteristics

File S4. Pairwise ANI (Average Nucleotide Identity) estimates and k-mer jaccard similarities between hybrid (long + short read) genome assemblies of each dataset (*Mtb*, *E. coli, and S. aureus).*

File S6. *Mtb* SV pan-genome graph (GFA). Built from H37Rv and 151 hybrid assemblies using the minigraph (v0.19) algorithm.

File S7. k-mer based analysis of SV node sequence composition and uniqueness within *Mtb* SV pan-genome graph

File S8. *Mtb*, *E. coli, and S. aureus* pan-genome estimates produced by all pipeline-parameter combinations evaluated.

File S9. Frequency of annotated feature type (CDS, pseudogene) estimated by PGAP & Bakta when applied to all 151 *Mtb* hybrid assembly sequences.
File S10. Table of manually identified differences in pseudogene annotation between PGAP & Bakta when applied to H37Rv reference genome sequence (H37Rv

File S11. Pseudogene annotation comparison visualization between the standard NCBI annotation, PGAP, and Bakta for the H37Rv reference sequence

File S5. Phylogeny of dataset of 151 *Mtb* isolates (newick format)

File S12. Phylogeny of dataset 50 *E. coli* isolates in (newick format)

File S13. Phylogeny of dataset of 68 *S. aureus* isolates in (newick format)

File S13. All genome assemblies and their respective genome annotations used in this work (*Mtb*, *E. coli, and S. aureus)*. This includes both hybrid (long + short read) and de novo short-read assemblies for each isolate.

Files S5, S6, S12, S13 are hosted on Zenodo due to their non-standard file type or large file size, at https://zenodo.org/records/10846276

Supplemental Results

SR1 - Comparison of short and long-read characteristics

We compared the assembly quality characteristics between the short-read and long read assemblies (**Table 1, Figure S1**). All complete assemblies consist of a single circular contig, while the short-read assemblies are consistently more fragmented with a median number of contigs of 116 (IQR: 106 - 132). The median genome size of the complete assemblies is 4,413 kb (4,407 kb - 4,421 kb). The cumulative length of the short-read assemblies are slightly lower than the complete genome assemblies, with a median length of 4,314 kb (IQR: 4,289 kb - 4,336 kb). Both technologies produced assemblies with a highly similar GC content, but the short-read assemblies varied slightly more: median GC content of the hybrid assemblies was 65.6%, IQR: 65.6%-65.6%, and for short-read assemblies the median GC content was 65.5%, IQR 65.5% - 65.6%. We found very high conservation in terms of the # of predicted ORFs for both short and complete assemblies. The median number of ORFs predicted for complete assemblies was 4074 (IQR: 4065 - 4088), while for short-read assemblies it was 4035 (IQR: 4022 - 4046). In summary, hybrid and short-read assembly characteristics were similar except that short-read assemblies systematically were less continuous, had lower cumulative length, and contained less predicted CDSs than complete assemblies.

SR2 - Identifying novel sequence relative to the H37Rv reference genome

Next, we wanted to evaluate what proportion of SV nodes in the graph represented sequences not present in the H37Rv reference genome. These SVs are of interest as they represent sequences that were lost in the ancestor to H37Rv but are present in other *Mtb* isolates. We found that only 160/2025 of evaluated SV nodes contained novel nucleotide content relative to H37Rv, with a cumulative length of 66,621 bp. Thus, only 5.5% of evaluated SV nodes within the graph represent novel sequence content relative to H37Rv. Thus, a majority of evaluated SV nodes in the graph are composed of either reconfiguration of already existing nucleotide content or deletions of regions that are present in the H37Rv reference genome.

The 160 SV nodes with novel sequence content relative to H37Rv were spread across 65 different bubble regions in the Mtb pan-genome. To focus our attention on larger SVs with novel sequences relative to H37Rv. From this list we were motivated to quantify the number of potentially large deletions (1 > kb) that occurred in the ancestor of the H37Rv strain. We generated a short list of 18 bubble regions that contained at least 1 kb of novel sequence relative to H37Rv (**Table S1**). The length of cumulative sequence that was novel relative to H37Rv ranged from 1153 bp to 7,900 bp per bubble region.

Notably, Bubble 193 matched the exact coordinates of a known structural variant within the Mtb population, Tbd1. The Tbd1 region is a deletion specific to lineages 2-4 of the *Mtb* population, and implicated in resistance to oxidative stress and hypoxia(1, 2). Our accurate detection of TbD1 serves as a positive control for the ability of our pan-genome graph approach to detect large structural differences between *Mtb* isolates. We further evaluated the graph representation of the Tbd1 deletion in terms of its genomic and phylogenetic context (**Figure S3**). Bubble 193 contains 3 different nodes and has three different observed configurations. The first observed configuration found within the graph is a complete

deletion of the expected Tbd1 sequence (ΔTbD1), and this allele was exclusively found in all lineage 2-4 isolates. The second observed configuration was a completely intact TbD1 sequence, found in lineages 1,5, & 6. This distribution of ΔTbD1 and +TbD1 alleles exactly matches prior knowledge of the phylogenetic distribution of this deletion across lineages 1-6. Interestingly, a third configuration of bubble 193 was observed for the single lineage 8 isolate within our collection. The lineage 8 isolate was found to have an IS6110 insertion element disrupting the *mmpL6* gene, presumably resulting in a loss of function. This supports likely convergent disruption of the *mmpL6* gene in the *Mtb* phylogeny, in the first case through deletion and second case through transposon insertion.

SR3 - Investigating differences between two bacterial genome annotation pipelines on H37Rv

Motivated by the consistent decrease in the predicted pan-genome size when using PGAP, we explored the differences between the PGAP and Bakta annotation pipelines. To compare these softwares on an identical genome sequence, we first annotated the H37Rv reference genome with both Bakta and PGAP and then compared these annotation results with the official H37Rv annotations (**Table S2**). Although both tools predicted approximately the same number of total genes, we found that PGAP annotated 138 less protein coding sequences (CDSs) than Bakta, but annotated 150 more pseudogenes in the H37Rv reference genome.

We manually inspected all pseudogenes in both annotations and found a high frequency of cases where a pseudogene identified by PGAP, is called as multiple CDSs by the Bakta software (**Supplemental File S11**). PGAP overall is more likely to call a sequence a pseudogene if it finds a suspect frameshift mutation is present in the nucleotide sequence of a gene sequence in a reference database. In contrast, Bakta is much more likely to call new CDSs in the position of a gene with a frameshift mutation. This pattern highlights a critical divergence in how the tools handle frameshifted or truncated sequences, which directly affects downstream pan-genome analyses.

SR4 - In short-read pan-genome analysis, the number of absent genes is often overestimated due to both incomplete assemblies and CDS annotation discrepancies

We next investigated how assembly completeness and continuity affects pan-genome inference when using short-read assemblies for analysis. In short-read pan-genome analysis, the number of absent genes is able to be overestimated due to both incomplete assemblies and/or annotation discrepancies. To understand the relative contribution of these two sources of bias, we analyzed CDS presence and DNA sequence presence in the outputs of Roary and Panaroo applied to short-read assemblies of Mtb. PPanGGolin and Pangene were excluded from this analysis because the softwares do not output the associated DNA sequences associated with each gene. We classified each case of gene absence into three categories: 1) Absence due to an incomplete SR assembly, where a gene absent in the short-read assembly can be found in the hybrid assembly, and 2) Absence due to CDS annotation discrepancy, where the CDS annotation of the gene is absent, but the gene can be identified at the nucleotide level, and 3) *bona fide* absence, where neither of the two previous criteria were met.

We found that 7%-13% of gene absences were associated with incomplete SR assembly, 44%-63% were associated with CDS annotation discrepancy (**Table 4**), while the minority were *bona fide* absences. Given this high proportion absences due to incomplete assembly or CDS annotation

discrepancy, we examined the relationship between the BUSCO completeness scores of each short-read assembly and each absence scenario. We categorized short-read assemblies based on their BUSCO scores: low (<99%, n=15) or high (>99%, n=136), and looked at its association with absence types, using our most conservative pan-genome estimate (**Figure S6, Methods**). Absences associated with incomplete assembly showed a significant increase in assemblies with low BUSCO scores (Mann-Whitney U test, p = 3.3e-9). However, the frequency of absences due to CDS annotation discrepancy was comparable between low and high BUSCO assemblies (Mann-Whitney U test, p = 0.12).

Supplemental Methods

Detailed *Mtb* hybrid assembly methods

A) PacBio Subreads Assembly & Polishing Pipeline

For all assemblies that were done with PacBio subreads, either from the RS II and Sequel II platforms, the following assembly processing was taken: All long reads (PacBio subreads) were assembled using Flye (v2.6). After assembly, Flye performed three rounds of iterative polishing of the genome assembly with long reads, producing a polished *de novo* long read assembly. If Flye identified the presence of a complete circular contig, Circlator (v1.5.5) was used to standardize the start of each assembly at the *DnaA* (*Rv0001*) locus. The paired-end Illumina WGS reads were trimmed with Trimmomatic (v0.39). Trimmed reads were aligned to the associated de novo long read assembly with BWA-MEM (v0.7.17). Duplicate reads were removed from the resulting alignments using PICARD (v2.22.5). Using the deduplicated alignments, Pilon (v1.23) was then used to correct SNSs and small INDELs in the de novo PacBio assembly. All relevant code, with exact run parameters, can be found in the `1.Mtb.Generate.HybridAsm.PacBioRSII.smk` script (Runnable as a Snakemake workflow).

B) ONT Assembly & Polishing Pipeline

For all assemblies made with Oxford Nanopore (Pore v9.4.1) the following assembly processing was taken: All ONT (v9.4.1) long reads ONT were assembled using Flye (v2.6). After the initial assembly, Flye performed three rounds of iterative polishing of the genome assembly with long reads, producing a polished de novo long read assembly. Next, the Medaka software was used to polish the de novo assembly with the ONT reads. If Flye identified the presence of a complete circular contig, Circlator (v1.5.5) was used to standardize the start of each assembly at the *DnaA* (*Rv0001*) locus. The paired-end Illumina WGS reads were trimmed with Trimmomatic (v0.39). Trimmed reads were aligned to the associated de novo long read assembly with BWA-MEM (v0.7.17). Duplicate reads were removed from the resulting alignments using PICARD (v2.22.5). Using the deduplicated alignments, Pilon (v1.23) was then used to correct SNSs and small INDELs in the de novo PacBio assembly. Next, the assembly was further polished with short reads using the PolyPolish (v0.5) pipeline. All relevant code, with exact run parameters, can be found in the `3.Mtb.Generate.HybridAsm.ONT9.4.1.smk` script (Runnable as a Snakemake workflow).

C) PacBio CCS (HiFi) Assembly & Polishing Pipeline:

For all assemblies generated from Sequel II CCS/HiFi reads the following assembly processing was taken: All PacBio HiFi reads were assembled using Flye (v2.9). After assembly, Flye performed three rounds of iterative polishing of the genome assembly with long reads, producing a polished de novo long read assembly. If Flye identified the presence of a complete circular contig, Circlator (v1.5.5) was used to standardize the start of each assembly at the *DnaA* (*Rv0001*) locus. The paired-end Illumina WGS reads were trimmed with Trimmomatic (v0.39). Trimmed reads were aligned to the associated de novo long

read assembly with BWA-MEM (v0.7.17). Duplicate reads were removed from the resulting alignments using PICARD (v2.22.5). Using the deduplicated alignments, Pilon (v1.23) was then used to correct SNSs and small INDELs in the de novo PacBio assembly. All relevant code, with exact run parameters, can be found in the `2.Mtb.Generate.HybridAsm.PacBioHiFi.smk` Snakemake workflow.

Evaluating sequence content of SV nodes within *Mtb* SV pan-genome graph

For the k-mer analysis of the graph, each node's sequence was broken down into all 31 bp subsequences. The mmh3 python library was used to generate the MurmurHash for all 31-mers within the dataset. Additionally, all k-mers were converted to their canonical k-mer such that a k-mer and its reverse complement would be given the same hash. For measurement of k-mer overlap between nodes and the rest of the graph the Jaccard Containment metric was used. Jaccard Containment is defined as the size of the union of two k-mer sets A and B divided by the length of set A. Put simply, the Jaccard containment is measured as the proportion of k-mers found within A that are also present in a second sequence, B.

For identification of SV nodes with unique sequence content, low k-mer overlap was defined as a k-mer Jaccard Containment of < 0.05. If a node was smaller than 31 bp it was excluded from this analysis. The ~500 SV nodes with length < 31 bp only have a cumulative length 6149 bp out of the 1,301,211 bp of SV nodes within the graph. As these short SV regions accounted for a small proportion (0.5%) of total SV node sequence, we expect that excluding them is unlikely to change our description of the Mtbc accessory genome.

To access classify nodes We compared the k-mer profile of each SV-node to all sequences annotated as related to "Insertion sequence elements & phages" in the H37Rv reference genome (Kapopoulou et al. 2011).

References

1. R. Brosch, *et al.*, A new evolutionary scenario for the Mycobacterium tuberculosis complex. *Proc. Natl. Acad. Sci. U. S. A.* **99**, 3684–3689 (2002).

2. D. Bottai, *et al.*, TbD1 deletion as a driver of the evolutionary success of modern epidemic Mycobacterium tuberculosis lineages. *Nat. Commun.* **11**, 684 (2020).

3. J. C. S. Ngabonziza, *et al.*, A sister lineage of the Mycobacterium tuberculosis complex discovered in the African Great Lakes region. *Nat. Commun.* **11**, 2917 (2020).

Supplemental Figures & Tables

Figure S1

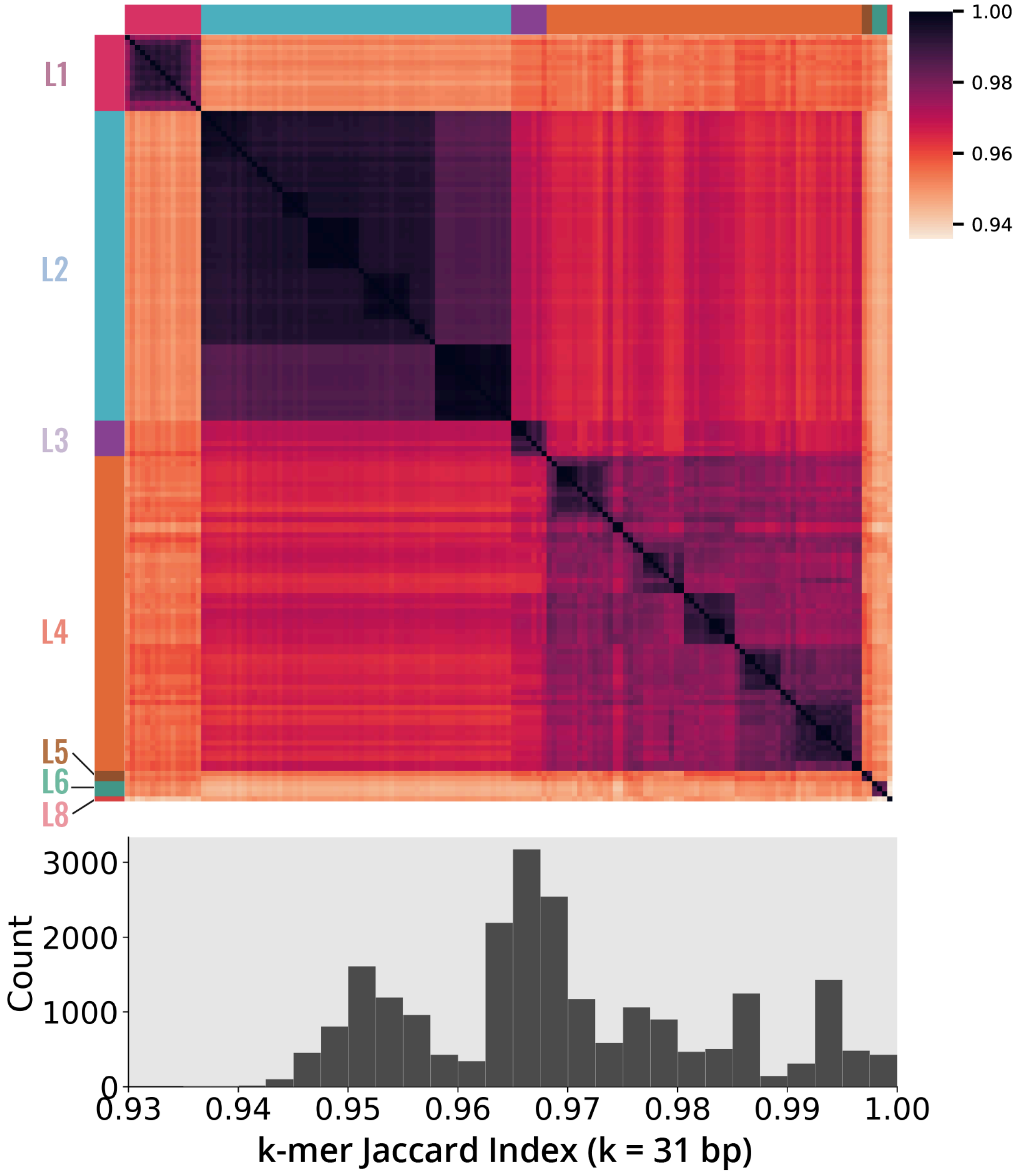

Figure S1. Overview of k-mer jaccard similarity across *Mtb* genomes. Heatmap and histogram of Jaccard Similarity of k-mers (k = 31 bp) across all pairs of complete *Mtb* genomes. The median k-mer jaccard similarity between all pairs was 0.97 (IQR: 0.96 - 0.98), while the most distant pair of genomes had a k-mer jaccard similarity of 0.94.

Figure S2

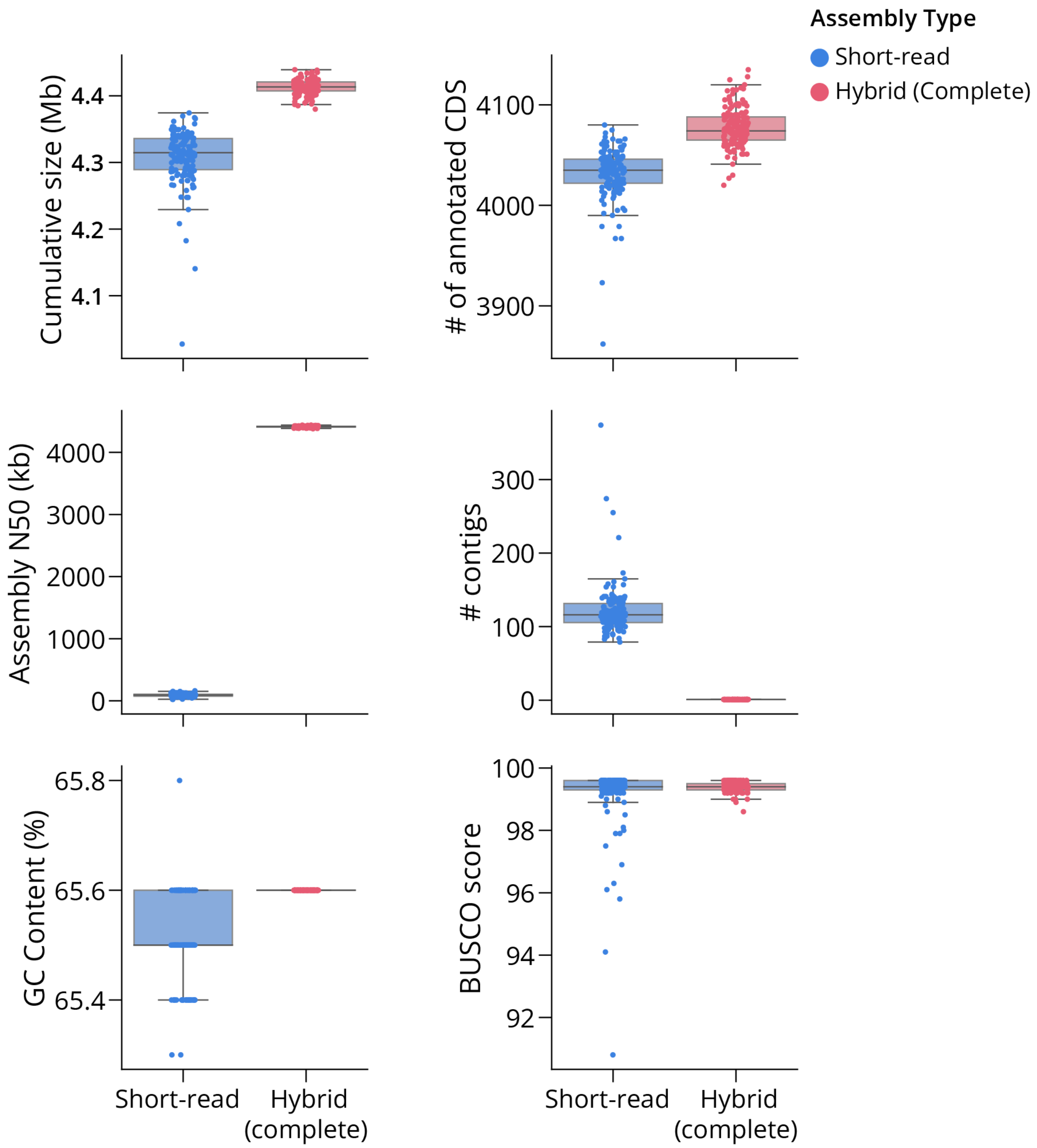

Figure S2. Comparison of assembly characteristics between complete assemblies and short-read *de novo* assemblies. Each of the 151 clinical *Mtb* isolates has both a complete hybrid assembly and a de novo short-read assembly available. The following characteristics were compared between the two assembly types: Cumulative assembly length, N50, GC Content, # of annotated CDSs with Bakta, # of total contigs, and BUSCO Completeness Score.

Table S1

Stat	Assembly Approach	Median w/ IQR
BUSCO Completeness Score	Hybrid (LR + SR)	99.4 (IQR: 99.3 - 99.5)
	Short read only	99.4 (IQR: 99.3 - 99.6)
Cumulative length	Hybrid (LR + SR)	4,413 kb (IQR: 4,407 - 4,421 kb)
	Short read only	4,314 kb (IQR: 4,289 - 4,336 kb)
# of contigs	Hybrid (LR + SR)	1 contigs (IQR: 1 - 1)
	Short read only	116 contigs (IQR: 106 - 132)
N50	Hybrid (LR + SR)	4,413 kb (IQR: 4,407 - 4,421 kb)
	Short read only	92 kb (IQR: 73 - 107 kb)
# of predicted CDSs (Bakta)	Hybrid (LR + SR)	4074 (IQR: 4065 - 4088)
	Short read only	4035 (IQR: 4022 - 4046)
GC Content	Hybrid (LR + SR)	65.6% (IQR: 65.6 - 65.6%)
	Short read only	65.5% (IQR: 65.5 - 65.6%)

Table S1. Assembly characteristics compared between complete & short read Mtb assemblies (N=151)

Table S2

Gene Functional Category (Based on MycoBrowser Annotations)	Cumulative length (bp) - SV Nodes w/ non-unique sequence content	# SV Nodes - w/ non-unique sequence content	Relative Percent of SV Nodes w/ non-unique sequence content (%)
insertion sequences & phage sequences	503748	346	39.46
PE/PPE Genes	167166	647	13.1
information pathways	4363	10	0.34
conserved hypotheticals	66735	111	5.23
cell wall and cell processes	42715	73	3.35
stable RNAs	0	0	0
intermediary metabolism and respiration	69088	105	5.41
regulatory proteins	13000	21	1.02
virulence, detoxification, adaptation	10554	7	0.83
lipid metabolism	51437	39	4.03
No Clear Classification	347744	666	27.24

Table S2. *Mtb* gene functional category breakdown associated with SV nodes with redundant (non-unique) sequence content.

Table S3

BubbleID	H37Rv coordinates	Overlapping H37Rv Genes	# SV Nodes	Cumulative SV Node Length	Cumulative length w/ LOW k-mer match to H37Rv
Bubble #31	334,243 - 338,669	pe-pgrs3, pe-pgrs4	70	12,630 bp	3,211 bp
Bubble #60	560425 - 561498	umaA, pcaA	2	2,074 bp	1,001 bp
Bubble #102	917629 918744	Rv0823c, desA1	4	2,210 bp	1,095 bp
Bubble #116	1,053,645 - 1,053,787	Rv0943c, Rv0944	2	4,541 bp	4,399 bp
Bubble #129	1181855 - 1215202	fadD14 - pe10	48	67,323 bp	1,697 bp
Bubble #193	1761789 - 1761789	mmpL6	3	3,511 bp	2,153 bp
Bubble #221	1986625 - 1999613	Rv1754c - Rv1765A	116	53,885 bp	7,500 bp
Bubble #255	2208005 - 2223187	yrbE3A - Rv1979c	26	29,094 bp	4,496 bp
Bubble #265	2268723 - 2268723	Rv2023A, Rv2024c	17	8,232 bp	5,518 bp
Bubble #362	3114093 - 3157869	Rv2807 - cobO	224	135,873 bp	1,232 bp
Bubble #380	3251646 - 3252957	ppsB	2	2,441 bp	1,130 bp
Bubble #396	3403272 - 3404591	ctaD	2	2,725 bp	1,406 bp
Bubble #404	3486249- 3487460	cyp141	4	3,722 bp	1,153 bp
Bubble #410	3501223 - 3501723	PPE50	13	1,859 bp	1,310 bp
Bubble #412	3528674 - 3529255	PPE53	6	5,517 bp	1,642 bp
Bubble #426	3709332 - 3711855	moaX - Rv3327	53	29,317 bp	4,495 bp
Bubble #430	3730530 - 3735813	PPE54	22	11,289 bp	1,342 bp
Bubble #476	3937189 - 3938416	ilvX	2	2,470 bp	1,243 bp

Table S3. 18 bubble regions identified with at least 1 kb of novel sequence relative to H37Rv. Each bubble identified has at least 1 kb of sequence from SV nodes that have low k-mer match to the H37Rv reference genome. Coordinates of each bubble are given relative to the H37Rv genome assembly. Bubble #193 overlaps exactly with the known coordinates of the 2.1 kb TbD1 deletion, which is implicated in increased resistance to oxidative stress and hypoxia(2). Bubble #116 overlaps with a 4.4 kb sequence identified to be exclusive to lineage 8 of the MTBC(3).

Figure S3

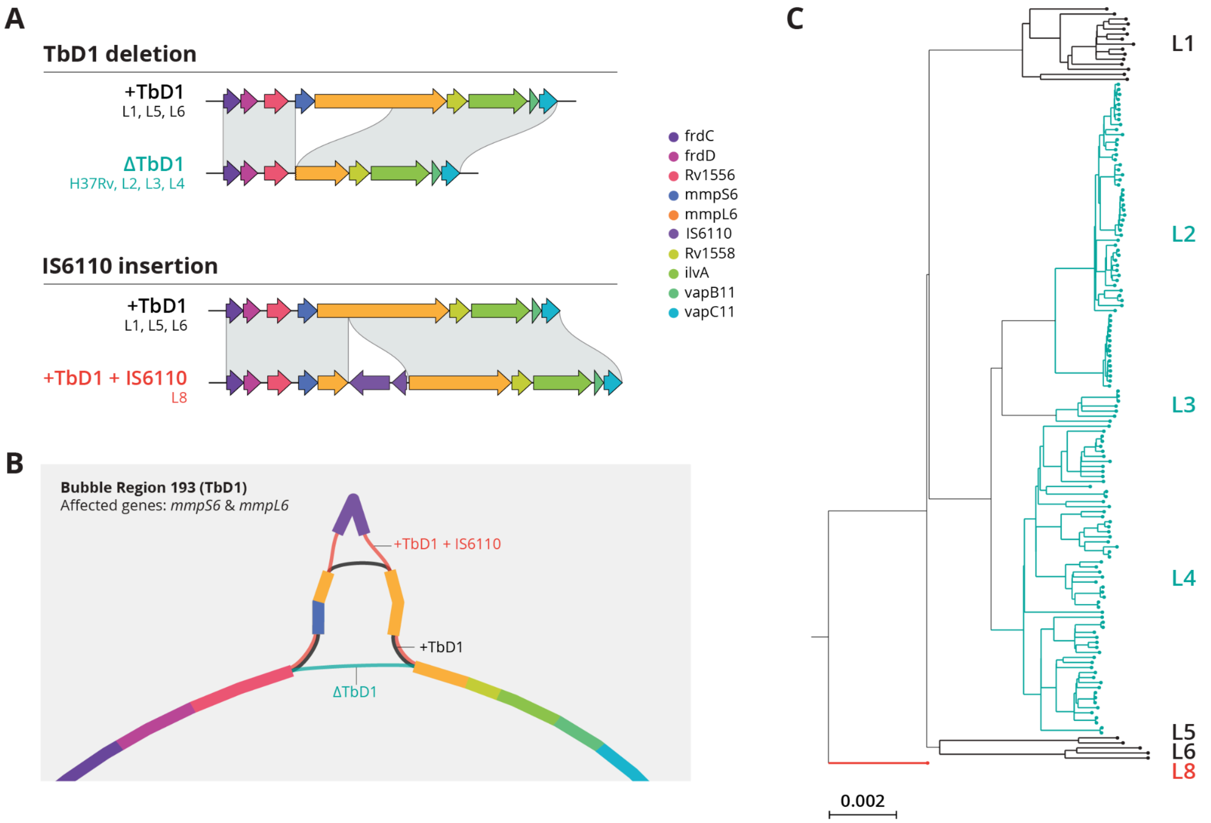

Figure S3. Evaluation of structural variation in TbD1 locus using a pan-genome graph. A) The TbD1 deletion refers to a deletion of the entire *mmpS6* gene and part of the *mmpL6* gene observed in lineages 2, 3 and 4 of the MTBC. Lineages 1, 5, and 6 contain the completely intact TbD1 locus (+TbD1). A third configuration of the locus is observed where an IS6110 transposable element has inserted in the mmpL6 gene (+TbD1- 6110), likely disrupting the protein function. **B)** Within the SV pan-genome graph, bubble region #193 represents these three configurations TbD1 locus. Bubble region #193 matches the exact coordinates of the known TbD1 deletion site in the H37Rv genome (NC_000962.3:1,761,789). **(C)** Mapping the presence of the three different configurations at the TbD1 locus onto the phylogeny of all isolates. There appears to be convergent loss of function of the mmpL6 gene in the *Mtb* phylogeny, one by deletion and the other by IS6110 insertion.

Table S4

	Bakta	PGAP	Mycobrowser	NCBI annotation (NC_000962.3)
**Protein coding**	4,073	3,967	4,018	3,906
**Pseudogenes**	6	296	13	30
**RNA genes**	70	49	142	68
**Total**	4,149	4,312	4,173	4,004

Table S3. Annotation type summary for H37Rv reference genome annotations.

Table S5

	Bakta	PGAP
**Protein coding (CDS)**	4,067 (IQR: 4,057 - 4,080)	3,919 (IQR: 3,900 - 3,930)
**Pseudogenes**	9 (IQR: 7 - 10)	376 (IQR: 339 - 396)
**RNA genes**	72 (IQR: 71 - 72)	49 (IQR: 0)
**Total**	4,147 (IQR: 4,138 - 4,160)	4,340 (IQR: 4,320 - 4,356)

Table S4. Summary table of feature types annotated by Bakta & PGAP across all 151 complete *Mtb* genome assemblies.

Figure S4

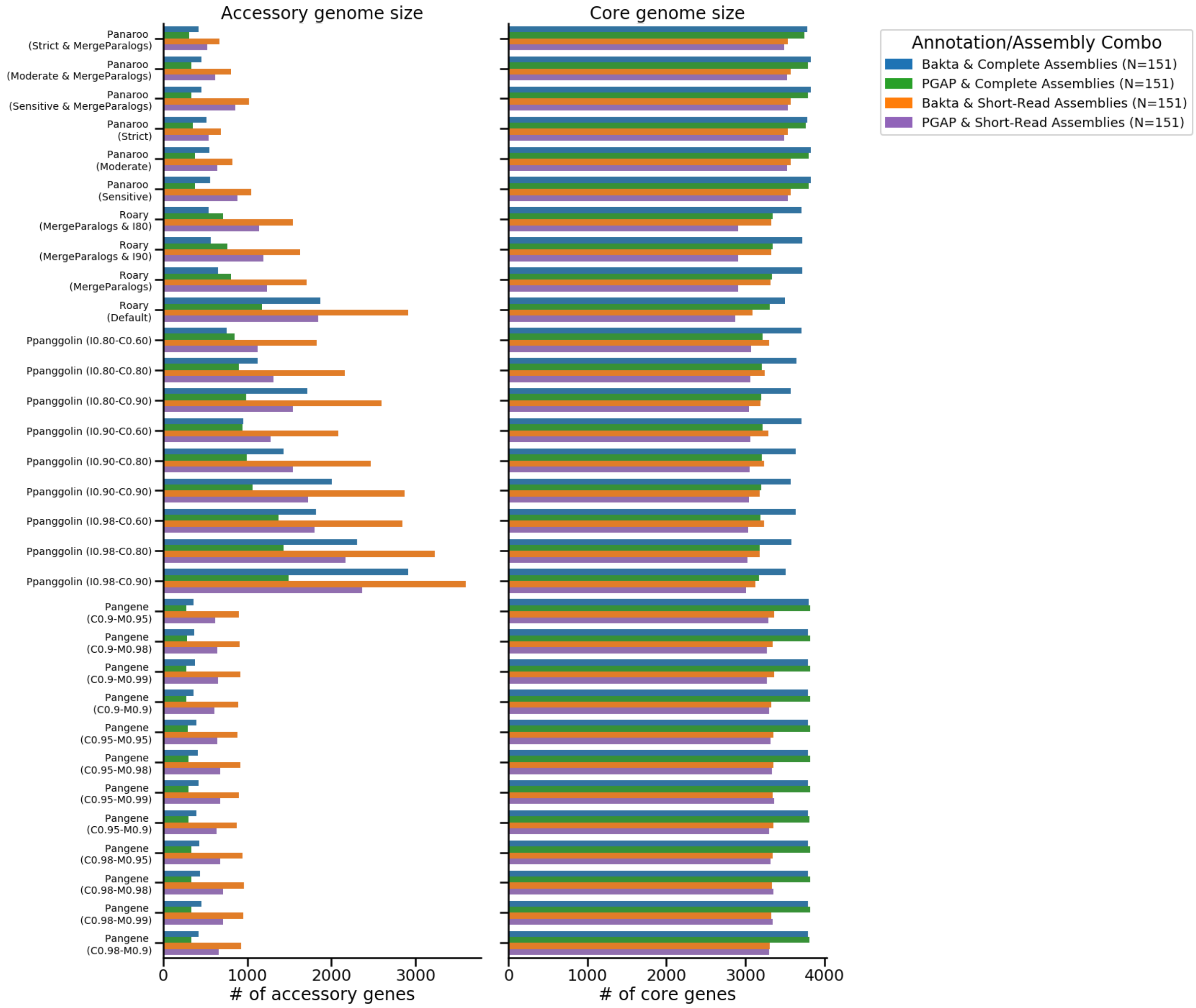

FIgure S4. ***Mtb*** **Pan-genome estimates for all individual pipeline-parameter combinations evaluated.**

Figure S5

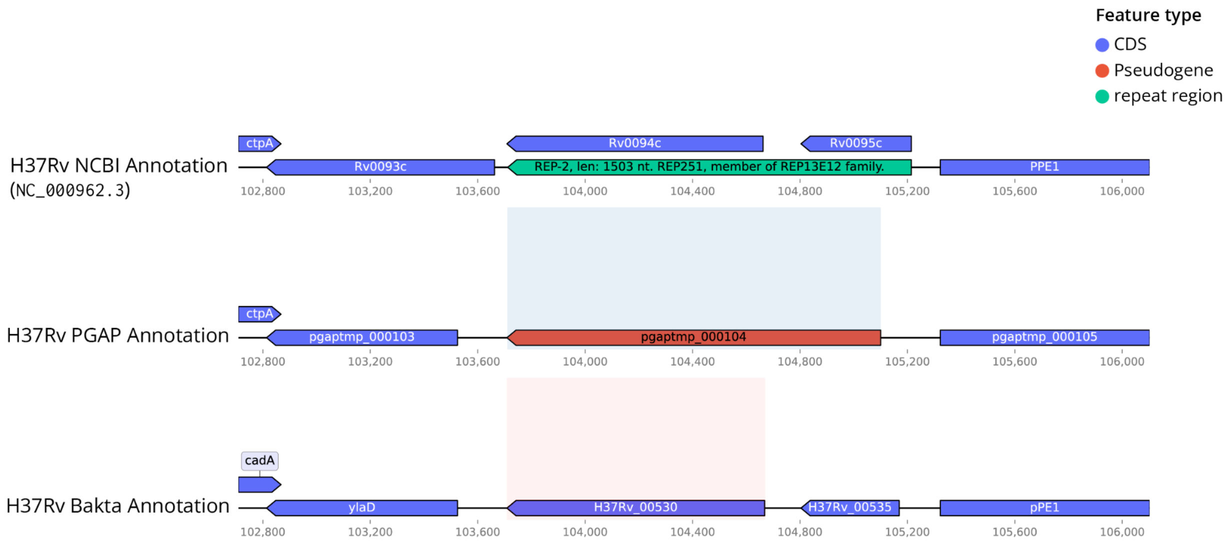

FIgure S5. Example of pseudogene annotated by PGAP in place of two distinct CDSs annotated by Bakta. In place of Rv0094c and Rv0095c, we observed that PGAP annotated these two ORFs as a single pseudogene. The official NCBI H37Rv reference and Bakta both infer this sequence to contain two separate CDSs. Supplemental file S11 contains information regarding all 37 split CDS vs pseudogene discrepancies manually identified between H37Rv's Bakta and PGAP annotations.

Table S6

Pipeline & Parameters	Total # absent genes	# absences associated with "CDS Annotation Discrepancy"	# Absences associated with "Incomplete SR assembly"
Panaroo - Strict & MergeParalogs (Short-read)	35,173	15606 (44.4%)	4514 (12.8%)
Panaroo - Moderate & MergeParalogs (Short-read)	51,869	24976 (48.2%)	6569 (12.7%)
Panaroo - Sensitive & MergeParalogs (Short-read)	83,674	40144 (48.0%)	9451 (11.3%)
Roary - MergeParalogs & I90 (Short-read)	151,288	93822 (62.0%)	10993 (7.3%)
Roary - MergeParalogs & I80 (Short-read)	141,774	89714 (63.3%)	10240 (7.2%)
Roary - MergeParalogs & I98 (Short-read)	161,268	98930 (61.4%)	12038 (7.5%)

Table S5. Sources of predicted gene absences with short-read based Mtb pan-genome analyses. Through comparison of each absent gene's nucleotide sequence to each Mtb isolates short-read and complete genome assembly, gene absences were identified that had signatures of "CDS annotation discrepancy" or "incomplete SR assembly". These alternative scenarios were compared against the total number of absent genes detected.

Figure S6

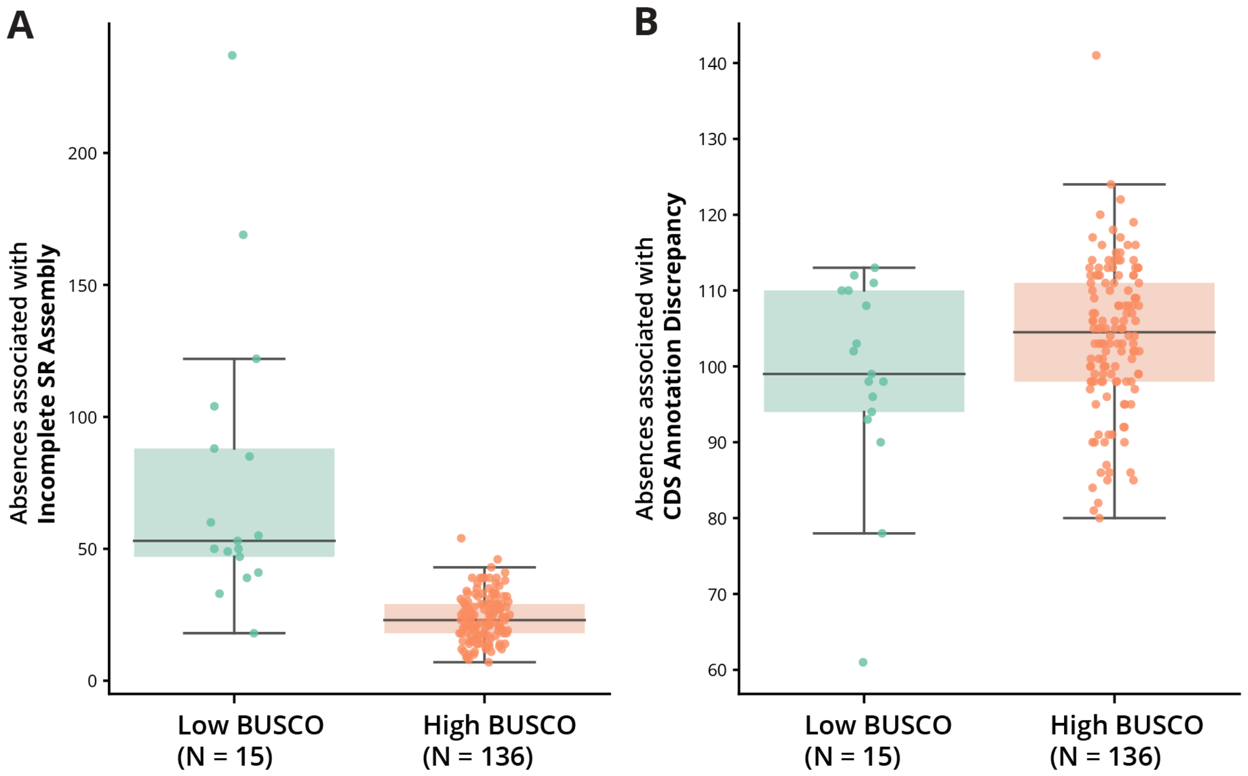

Figure S6. Evaluating scenarios contributing to inferred absences in short-read assemblies between low and high BUSCO completeness scores. All SR assemblies were classified as either low BUSCO assemblies (BUSCO < 0.99, N = 15) or high BUSCO assemblies (BUSCO >= 0.99, N = 136). Analysis of the frequency of absences related to "incomplete SR assembly" and "CDS annotation discrepancy" between low and high BUSCO assemblies was performed using the estimates generated by using Bakta annotation & Panaroo (`--clean-mode strict --merge_paralogs`). **A)** Boxplot of absences associated with "incomplete SR assembly" for both low BUSCO and high BUSCO SR assemblies. A low BUSCO SR assemblies were associated with a significant increase in inferred absences related to incomplete assembly (Mann-Whitney U test, p = 3.3e-9) **B)** Boxplot of absences associated with "CDS annotation discrepancies" for both low BUSCO and high BUSCO SR assemblies. No significant difference in the frequency of absences associated with "CDS annotation discrepancy" was observed between low and high BUSCO assemblies (Mann-Whitney U test, p = 0.12).

Figure S7

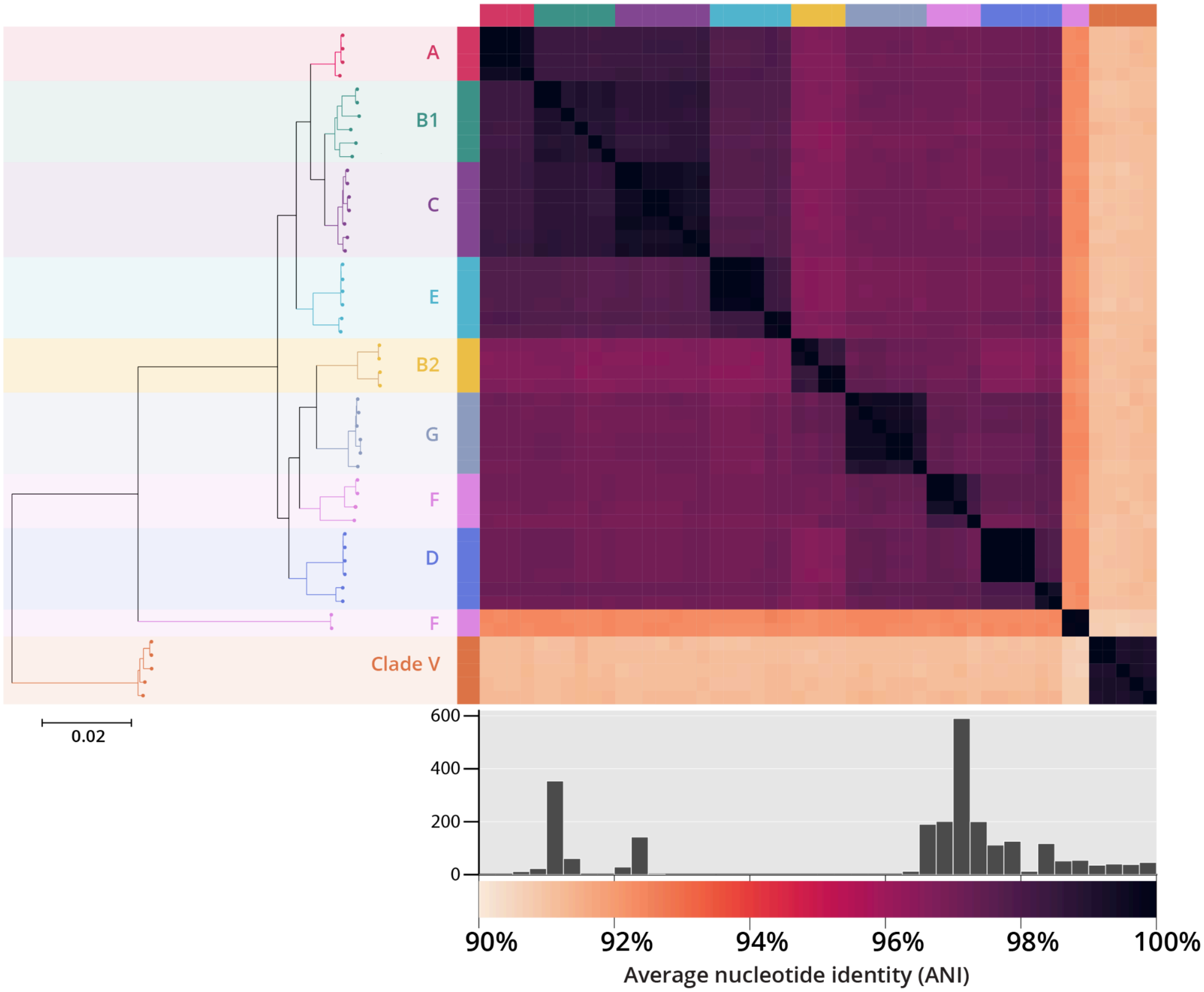

Figure S7. Overview of selected *E. coli* isolates from Shaw-2021. 50 *E. coli* isolates from across 9 phylogroup classifications were selected as a representative dataset from Shaw et. al. 2021. A heatmap of estimated ANI values within the population is displayed with the core genome phylogeny displayed to the left. Each genome is colored by *E. coli* phylotype classifications identified in Shaw et. al. FastANI was used to estimate ANI between all pairs of genomes. The histogram of all pairwise ANI estimates is shown below. The median ANI across all pairs was 97.1% and ANI ranged from 90.6% to 100%.

Figure S8

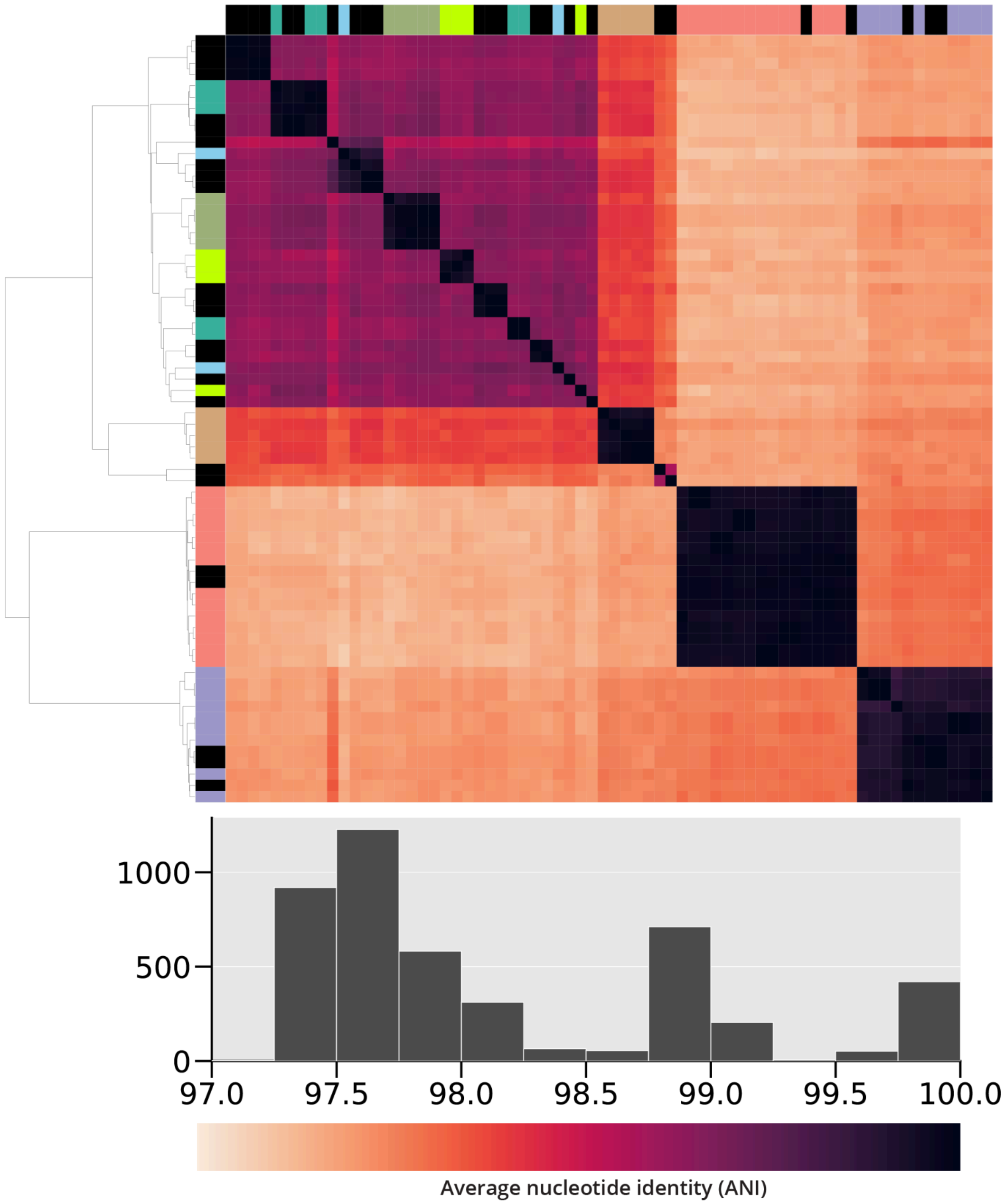

Figure S8. Overview of selected *S. aureus* isolates from Houtak-2023. 68 *S. aureus* isolates from across 9 Strain Type (ST) classifications were selected as a representative dataset of clinical *S. aureus* genomes from Houtak et. al. 2023. A heatmap of estimated pairwise average nucleotide identity values within the population is displayed. Each genome is colored by *S. aureus* strain type classifications identified in Shaw et. al 2023. FastANI was used to estimate ANI between all pairs of genomes. The histogram of all pairwise ANI estimates is shown below. The median ANI across all pairs was 97.1% and ANI ranged from 97.2% to 100%.

Table S6

Assembly type	Pipeline	Parameters	Total # absent genes	# absences associated with "CDS Annotation Discrepancy"
Complete	Panaroo	Strict & MergeParalogs	33046	10784 (32.6%)
Complete	Panaroo	Moderate & MergeParalogs	36487	12852 (35.2%)
Complete	Panaroo	Sensitive & MergeParalogs	36625	12976 (35.4%)
Complete	Roary	MergeParalogs & Identity 98 (Default)	64856	35226 (54.3%)
Complete	Roary	MergeParalogs & Identity 90	54830	29292 (53.4%)
Complete	Roary	MergeParalogs & Identity 80	51372	28144 (54.8%)
Short-read	Panaroo	Strict & MergeParalogs	35173	15606 (44.4%)
Short-read	Panaroo	Moderate & MergeParalogs	51869	24976 (48.2%)
Short-read	Panaroo	Sensitive & MergeParalogs	83674	40144 (48.0%)
Short-read	Roary	MergeParalogs & Identity 98 (Default)	161268	98930 (61.3%)
Short-read	Roary	MergeParalogs & Identity 90	151288	93822 (62.0%)
Short-read	Roary	MergeParalogs & Identity 80	141774	89714 (63.3%)

Table S6. Frequency of CDS Annotation discrepancies associated with absent gene calls in Mtb pan-genome analyses

Table S7

Assembly type	Pipeline	Parameters	Total # absent genes	# absences associated with "CDS Annotation Discrepancy"
Complete	Panaroo	Strict & MergeParalogs	481901	5915 (1.2%)
Complete	Panaroo	Moderate & MergeParalogs	492056	6050 (1.2%)
Complete	Panaroo	Sensitive & MergeParalogs	511206	6798 (1.3%)
Complete	Roary	MergeParalogs & Identity 98 (Default)	651347	9102 (1.4%)
Complete	Roary	MergeParalogs & Identity 90	506502	1565 (0.3%)
Complete	Roary	MergeParalogs & Identity 80	506502	1565 (0.3%)
Short-read	Panaroo	Strict & MergeParalogs	406594	4523 (1.1%)
Short-read	Panaroo	Moderate & MergeParalogs	435712	5869 (1.3%)
Short-read	Panaroo	Sensitive & MergeParalogs	536646	10516 (2.0%)
Short-read	Roary	MergeParalogs & Identity 98 (Default)	654563	10412 (1.6%)
Short-read	Roary	MergeParalogs & Identity 90	511437	2905 (0.6%)
Short-read	Roary	MergeParalogs & Identity 80	437116	2419 (0.6%)

Table S7. Frequency of CDS Annotation discrepancies associated with absent gene calls in E. coli pan-genome analyses.

Table S8

Assembly type	Pipeline	Parameters	Total # absences	# absences associated with "CDS Annotation Discrepancy"
Complete	Panaroo	Strict & MergeParalogs	105466	2405 (2.3%)
Complete	Panaroo	Moderate & MergeParalogs	107110	2458 (2.3%)
Complete	Panaroo	Sensitive & MergeParalogs	114480	2628 (2.3%)
Complete	Roary	MergeParalogs & Identity 98 (Default)	101654	566 (0.6%)
Complete	Roary	MergeParalogs & Identity 90	123845	863 (0.7%)
Complete	Roary	MergeParalogs & Identity 80	168980	6600 (3.9%)
Short-read	Panaroo	Strict & MergeParalogs	86031	1727 (2.0%)
Short-read	Panaroo	Moderate & MergeParalogs	90964	2152 (2.4%)
Short-read	Panaroo	Sensitive & MergeParalogs	119413	3238 (2.7%)
Short-read	Roary	MergeParalogs & Identity 98 (Default)	103772	487 (0.5%)
Short-read	Roary	MergeParalogs & Identity 90	124807	777 (0.6%)
Short-read	Roary	MergeParalogs & Identity 80	167868	6174 (3.7%)

Table S8. Frequency of CDS Annotation discrepancies associated with absent gene calls in S. aureus pan-genome analyses

Table S9

Assembly type	Pipeline	Parameters	Adjustment	# total size	# core	# accessory	ΔTotal	ΔCore	ΔAccessory
Hybrid	Panaroo	Strict & MergeParalogs	Original	4200	3779	421	-139	13	-152
			panqc	4061	3792	269			
Hybrid	Panaroo	Moderate & MergeParalogs	Original	4280	3825	455	-155	16	-171
			panqc	4125	3841	284			
Hybrid	Panaroo	Sensitive & MergeParalogs	Original	4281	3825	456	-156	16	-172
			panqc	4125	3841	284			
Hybrid	Roary	MergeParalogs & Identity 98 (Default)	Original	4366	3716	650	-187	133	-320
			panqc	4179	3849	330			
Hybrid	Roary	MergeParalogs & Identity 90	Original	4293	3723	570	-154	108	-262
			panqc	4139	3831	308			
Hybrid	Roary	MergeParalogs & Identity 80	Original	4252	3714	538	-138	100	-238
			panqc	4114	3814	300			
Short-read	Panaroo	Strict & MergeParalogs	Original	4211	3537	674	-158	49	-207
			panqc	4053	3586	467			
Short-read	Panaroo	Moderate & MergeParalogs	Original	4382	3574	808	-249	59	-308
			panqc	4133	3633	500			
Short-read	Panaroo	Sensitive & MergeParalogs	Original	4600	3576	1024	-431	70	-501
			panqc	4169	3646	523			
Short-read	Roary	MergeParalogs & Identity 98 (Default)	Original	5025	3318	1707	-657	297	-954
			panqc	4368	3615	753			
Short-read	Roary	MergeParalogs & Identity 90	Original	4956	3326	1630	-625	280	-905
			panqc	4331	3606	725			
Short-read	Roary	MergeParalogs & Identity 80	Original	4866	3324	1542	-584	266	-850
			panqc	4282	3590	692			

Table S9. Pan-genome size estimates for 151 *Mtb* isolates before and after panqc adjustment. *Mtb* pan-genome statistics (Total, Core, and Accessory genes) were quantified before and after panqc adjustment. The panqc pipeline was run with default settings where the minimum query coverage (90%) and length (90%) for gene rediscovery at the nucleotide level. Additionally, a k-mer similarity cutoff of 0.8 was used for identifying nucleotide similarity clusters.

Table S10

Assembly type	Pipeline	Parameters	Adjustment	# total size	# core	# accessory	ΔTotal	ΔCore	ΔAccessory
Hybrid	Panaroo	Strict & MergeParalogs	Original	14410	3035	11375	-93	6	-99
			panqc (Default)	14317	3041	11276			
Hybrid	Panaroo	Moderate & MergeParalogs	Original	14641	3036	11605	-95	6	-101
			panqc (Default)	14546	3042	11504			
Hybrid	Panaroo	Sensitive & MergeParalogs	Original	15032	3036	11996	-115	6	-121
			panqc (Default)	14917	3042	11875			
Hybrid	Roary	MergeParalogs & Identity 98 (Default)	Original	17783	2484	15299	-46	19	-65
			panqc (Default)	17737	2503	15234			
Hybrid	Roary	MergeParalogs & Identity 90	Original	14851	2915	11936	-21	5	-26
			panqc (Default)	14830	2920	11910			
Hybrid	Roary	MergeParalogs & Identity 80	Original	14851	2915	11936	-21	5	-26
			panqc (Default)	14830	2920	11910			
Short-read	Panaroo	Strict & MergeParalogs	Original	12836	2834	10002	-44	7	-51
			panqc (Default)	12792	2841	9951			
Short-read	Panaroo	Moderate & MergeParalogs	Original	13462	2834	10628	-71	8	-79
			panqc (Default)	13391	2842	10549			
Short-read	Panaroo	Sensitive & MergeParalogs	Original	15524	2834	12690	-202	8	-210
			panqc (Default)	15322	2842	12480			
Short-read	Roary	MergeParalogs & Identity 98 (Default)	Original	17825	2314	15511	-60	31	-91
			panqc (Default)	17765	2345	15420			
Short-read	Roary	MergeParalogs & Identity 90	Original	14933	2711	12222	-37	20	-57
			panqc (Default)	14896	2731	12165			
Short-read	Roary	MergeParalogs & Identity 80	Original	13420	2825	10595	-20	20	-40
			panqc (Default)	13400	2845	10555			

Table S10. Pan-genome size estimates for 50 *E. coli* isolates before and after panqc adjustment. *E. coli* pan-genome statistics (Total, Core, and Accessory genes) were quantified before and after panqc adjustment. The panqc pipeline was run with default settings where the minimum query coverage (90%) and length (90%) for gene rediscovery at the nucleotide level. Additionally, a k-mer similarity cutoff of 0.8 was used for identifying nucleotide similarity clusters.

Figure S9

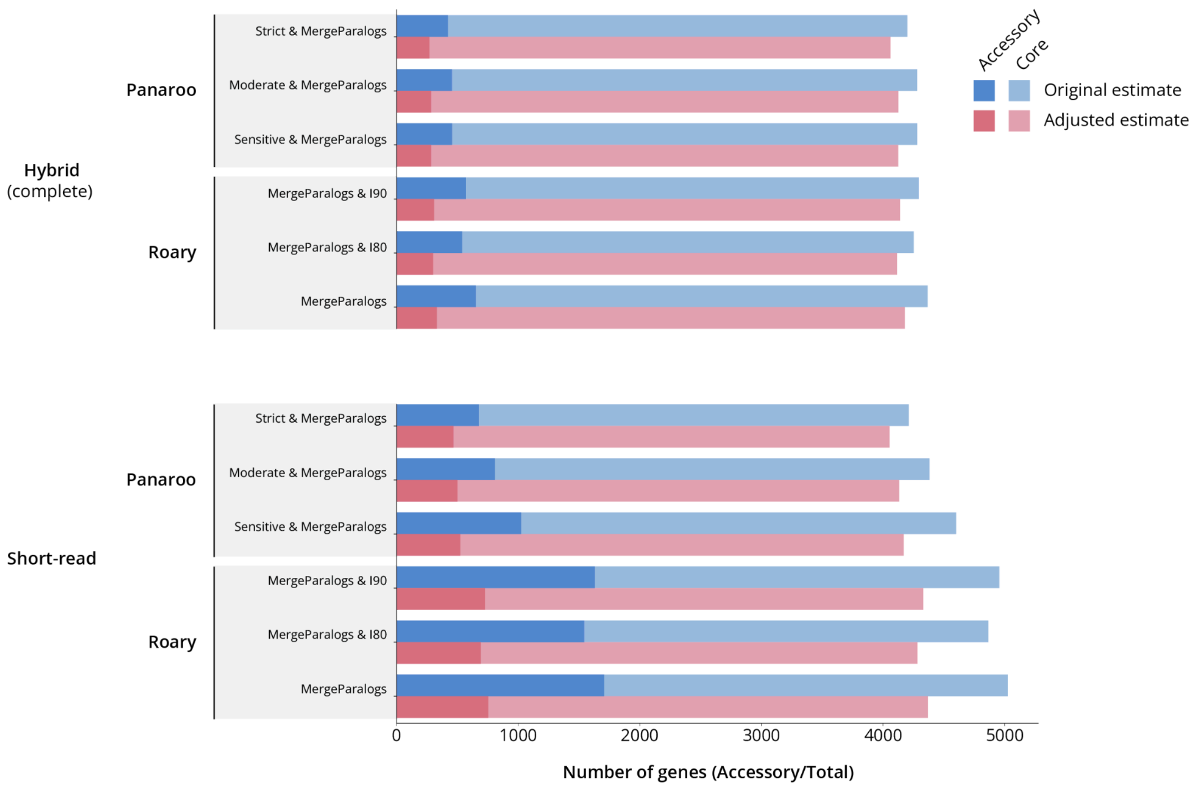

Figure S9. *Mtb* pan-genome estimates before and after panqc correction. The panqc pipeline was run on the pan-genome results for all Panaroo and Roary analyses that merged paralogs (--mergeparalogs). Core and accessory gene counts before and after panqc correction are shown in a stacked barplot. The solid color represents the accessory genome, while the transparent section of the bar represents the number of core genes inferred. The absolute bar height represents the total pan-genome size.

Figure S10

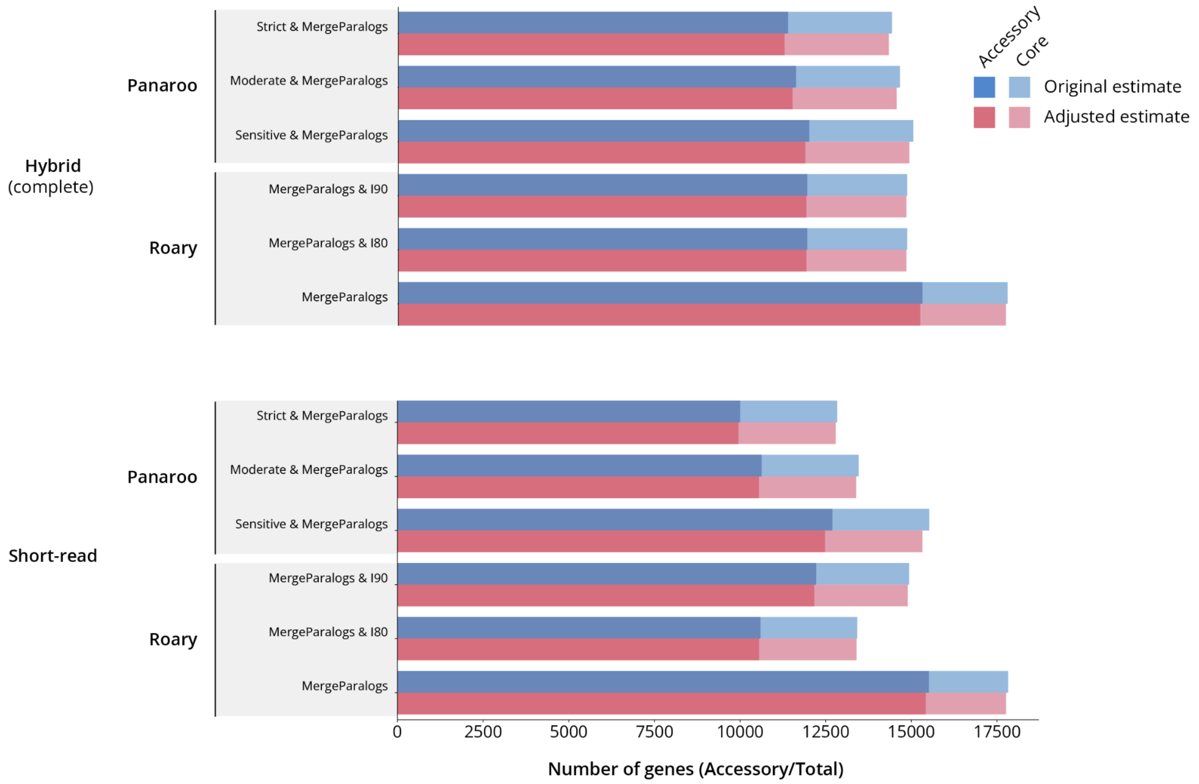

Figure S10. *E. coli* pan-genome estimates before and after panqc correction. The panqc pipeline was run on the pan-genome results for all Panaroo and Roary analyses that merged paralogs (--mergeparalogs). Core and accessory gene counts before and after panqc correction are shown in a stacked barplot. The solid color represents the accessory genome, while the transparent section of the bar represents the number of core genes inferred. The absolute bar height represents the total pan-genome size.

Table S11

**Assembly type**	**Pipeline**	**Parameters**	# nucleotide similarity clusters	# genes merged	Mean # of genes per cluster
Complete	Panaroo	Strict & MergeParalogs	111	250	2.25
Complete	Panaroo	Moderate & MergeParalogs	120	275	2.29
Complete	Panaroo	Sensitive & MergeParalogs	120	276	2.3
Complete	Roary	MergeParalogs & Identity 98 (Default)	139	347	2.5
Complete	Roary	MergeParalogs & Identity 90	107	272	2.54
Complete	Roary	MergeParalogs & Identity 80	91	235	2.58
Short-read	Panaroo	Strict & MergeParalogs	75	233	3.11
Short-read	Panaroo	Moderate & MergeParalogs	102	351	3.44
Short-read	Panaroo	Sensitive & MergeParalogs	151	582	3.85
Short-read	Roary	MergeParalogs & Identity 98 (Default)	393	1074	2.73
Short-read	Roary	MergeParalogs & Identity 90	372	1017	2.73
Short-read	Roary	MergeParalogs & Identity 80	352	953	2.71

Table S11. Summary of panqc nucleotide similarity clustering of *Mtb* pan-genome estimates. For each estimate analyzed with panqc, the following relevant statistics are shown after nucleotide similarity clustering using a minimum 31-mer overlap (maximum Jaccard Similarity observed) of 0.8: 1) The number of total nucleotide similarity (NS) clusters identified, 2) total number of genes merged into NS clusters, and 3) the mean number of genes belonging to each NS cluster.

Table S12

**Assembly type**	**Pipeline**	**Parameters**	# nucleotide similarity clusters	# genes merged	Mean # of genes per cluster
Complete	Panaroo	Strict & MergeParalogs	78	171	2.19
Complete	Panaroo	Moderate & MergeParalogs	79	174	2.2
Complete	Panaroo	Sensitive & MergeParalogs	90	205	2.28
Complete	Roary	MergeParalogs & Identity 98 (Default)	49	104	2.12
Complete	Roary	MergeParalogs & Identity 90	24	49	2.04
Complete	Roary	MergeParalogs & Identity 80	24	49	2.04
Short-read	Panaroo	Strict & MergeParalogs	35	79	2.26
Short-read	Panaroo	Moderate & MergeParalogs	57	128	2.25
Short-read	Panaroo	Sensitive & MergeParalogs	146	348	2.38
Short-read	Roary	MergeParalogs & Identity 98 (Default)	60	129	2.15
Short-read	Roary	MergeParalogs & Identity 90	38	80	2.11
Short-read	Roary	MergeParalogs & Identity 80	22	45	2.05

Table S12. Summary of NRC nucleotide similarity clustering of *E. coli* pan-genome estimates. For each estimate analyzed with panqc, the following relevant statistics are shown after nucleotide similarity clustering using a minimum 31-mer overlap (maximum Jaccard Similarity observed) of 0.8: 1) The number of total nucleotide similarity (NS) clusters identified, 2) total number of genes merged into NS clusters, and 3) the mean number of genes belonging to each NS cluster.
